# Tau Accumulation Induces Microglial State Alterations in Alzheimer's Disease Model Mice

**DOI:** 10.1523/ENEURO.0260-24.2024

**Published:** 2024-12-04

**Authors:** Kenichi Nagata, Shoko Hashimoto, Daisuke Joho, Ryo Fujioka, Yukio Matsuba, Misaki Sekiguchi, Naomi Mihira, Daisuke Motooka, Yu-Chen Liu, Daisuke Okuzaki, Masataka Kikuchi, Shigeo Murayama, Takaomi C. Saido, Hiroshi Kiyama, Hiroki Sasaguri

**Affiliations:** ^1^Department of Functional Anatomy and Neuroscience, Nagoya University, Graduate School of Medicine, Aichi 466-8550, Japan; ^2^Laboratory for Proteolytic Neuroscience, RIKEN Center for Brain Science, Saitama 351-0198, Japan; ^3^Pioneering Research Division, Medical Innovation Research Center, Shiga University of Medical Science, Shiga 520-2192, Japan; ^4^Dementia Pathophysiology Collaboration Unit, RIKEN Center for Brain Science, Saitama 351-0198, Japan; ^5^Genome Information Research Center, Research Institute for Microbial Diseases, Osaka University, Osaka 565-0871, Japan; ^6^Integrated Frontier Research for Medical Science Division, Institute for Open and Transdisciplinary Research Initiatives, Osaka University, Osaka 565-0871, Japan; ^7^Single Cell Genomics, Human Immunology, WPI Immunology Frontier Research Center, Osaka University, Osaka 565-0871, Japan; ^8^Department of Computational Biology and Medical Sciences, Graduate School of Frontier Science, The University of Tokyo, Chiba 277-0882, Japan; ^9^Department of Molecular Genetics, Brain Research Institute, Niigata University, Niigata 951-8585, Japan; ^10^Department of Neuropathology, Tokyo Metropolitan Institute of Gerontology, Tokyo 173-0015, Japan; ^11^Brain Bank for Neurodevelopmental, Neurological and Psychiatric Disorders, Molecular Research Center for Children’s Mental Development, United Graduate School of Child Development, Osaka University, Osaka 565-0871, Japan; ^12^Shijonawate Gakuen University, Osaka 574-0001, Japan

**Keywords:** Alzheimer's disease, amyloid-β, microglia, single-cell RNA-seq, tau

## Abstract

Unique microglial states have been identified in Alzheimer's disease (AD) model mice and postmortem AD brains. Although it has been well documented that amyloid-β accumulation induces the alteration of microglial states, the relationship between tau pathology and microglial states remains incompletely understood because of a lack of suitable AD models. In the present study, we generated a novel AD model mouse by the intracerebral administration of tau purified from human brains with primary age-related tauopathy into *App* knock-in mice with humanized tau. Immunohistochemical analyses revealed that Dectin-1-positive disease-associated microglia were increased in the AD model mice after tau accumulation in the brain. We then performed single-nucleus RNA sequencing on the AD model mice to evaluate the differences in microglial states with and without tau propagation and accumulation. By taking advantage of spatial transcriptomics and existing single-cell RNA sequencing datasets, we showed for the first time that tau propagation and accumulation induce a disease-associated microglial phenotype at the expense of an age-related nonhomeostatic counterpart (namely, white matter-associated microglia) in an AD model mouse brain. Future work using spatial transcriptomics at single-cell resolution will pave the way for a more appropriate interpretation of microglial alterations in response to tau pathology in the AD brain.

## Significance Statement

We generated a novel Alzheimer's disease model mouse showing humanized tau accumulation and propagation after amyloid-β accumulation. Immunohistochemical analyses revealed that disease-associated microglia were increased in the model mice after tau accumulation in the brain. Using single-cell RNA-seq and spatial transcriptomics, we showed for the first time that tau propagation and accumulation induce a disease-associated microglial phenotype at the expense of an age-related nonhomeostatic counterpart in the model mouse brain.

## Introduction

Alzheimer's disease (AD) is a neurodegenerative disorder that is pathologically characterized by amyloid-β deposits and tau accumulation (Jucker and Walker, 2013). Amyloid-β accumulates in large areas of the brain as early as 20 years before the appearance of clinical symptoms and is followed by tau abnormalities (Jack et al., 2013). However, Braak et al. have reported that AD-like tau pathology is present in the transentorhinal region as early as at 20 years of age—and above the age of 60 years, most people have AD-like tau pathology in the transentorhinal region, entorhinal region proper, and hippocampal formation, independent of their underlying diseases (Braak et al., 2011). Tau pathology in these regions seems to remain relatively restricted if there is no or little amyloid pathology in the brain; recently, this condition was pathologically defined as primary age-related tauopathy (PART). Interestingly, the biochemical and pathological features of PART tau are similar to those of AD tau (Crary et al., 2014). Moreover, a recent cryo-electron microscopic analysis revealed that tau filament structures are identical between AD and PART (Shi et al., 2021). These findings indicate that extensive amyloid pathology may induce the spreading of tau pathology from the limbic region and suggest that PART tau might be the first seed of AD tau pathology. This assumption is supported by a longitudinal positron emission tomography study for amyloid and tau in aging and AD subjects (Sanchez et al., 2021).

Recent advances in single-cell RNA sequencing (RNA-seq) have made it possible to characterize detailed cellular responses in pathological conditions. To date, microglia—a tissue-resident macrophage in the brain—is the most well-characterized cell type in the AD brain. Unique microglial states have been recently identified using single-cell RNA-seq in various genetically modified AD model mice, such as 5XFAD (Keren-Shaul et al., 2017; Zhou et al., 2020), APPPS1 (Van Hove et al., 2019), and *App^NL-G-F^* mice (Frigerio et al., 2019). These disease-associated microglia (DAMs) highly express many genes that are barely detectable in their homeostatic counterparts. With amyloid-β accumulation, the DAM phenotype arises, and DAMs then surround amyloid plaques in an apolipoprotein E (APOE)- and triggering receptor expressed on myeloid cells 2 (TREM2)-dependent manner (Keren-Shaul et al., 2017; Frigerio et al., 2019); both APOE and TREM2 are well-known AD risk genes (Pimenova et al., 2018). A marked reduction in DAM number has been reported in the APOE-deficient AD model, thereby changing the amyloid load in the brain (Frigerio et al., 2019). More recent studies have shown that DAMs play an essential role in inhibiting amyloid-β accumulation via their phagocytic activity (Huang et al., 2021; S. T. Wang et al., 2022). Although DAM studies have continued to improve our understanding of microglia in the AD brain, it is important to note that DAMs do not comprise a single population but are rather a unique population in each situation (Paolicelli et al., 2022). Furthermore, given that many AD models that have been used to detect DAM are simple amyloid models, it has yet to be resolved whether microglial states are sustained from the amyloid-β accumulation stage to the subsequent stage of both amyloid-β and tau accumulation.

In the current study, we performed immunohistochemistry and single-nucleus RNA-seq on a recently established humanized AD model (Hashimoto et al., 2019; Saito et al., 2019) with or without the intracranial administration of PART tau, to investigate the effects of tau accumulation on microglial cellular states in the brain. Moreover, subsequent multimodal integrative analyses and reanalyses using existing single-cell RNA-seq datasets enabled us to extract the microglial changes caused by tau pathology and distinguish different microglial subpopulations.

## Materials and Methods

### Animals

All animal experiments were performed according to the animal experimental guidelines of RIKEN. This research was approved by the ethics committee of RIKEN and the Tokyo Metropolitan Institute for Geriatrics and Gerontology. The study was performed in accordance with the ethical standards as laid down in the 1964 Declaration of Helsinki and its later amendments or comparable ethical standards. *App^NL-G-F^/MAPT* double knock-in (dKI) mice (Hashimoto et al., 2019; Saito et al., 2019) were used as AD model mice. These *App^NL-G-F^*/*MAPT* dKI mice were generated by crossing *App^NL-G-F^* with *MAPT* knock-in mice. *App^NL-G-F^* is a knock-in AD model in which the gene sequence encoding amyloid-β (i.e., *App*) is humanized using three distinct pathogenic AD mutations (Saito et al., 2014). The name of the model includes the amino acid sequences of the three *App* mutations: Swedish mutation (K670N/N671L; Mullan et al., 1992), Arctic mutation (E693G; Kamino et al., 1992; Nilsberth et al., 2001), and Iberian mutation (I716F; Lichtenthaler et al., 1999; Guerreiro et al., 2010). The *MAPT* knock-in mouse is a genetically modified mouse in which the *Mapt* genomic locus (encoding microtubule-associated protein tau) is humanized to recapitulate the three- and four-repeat tau splicing forms.

### Preparation of human brain-derived tau

Human brain samples were kindly provided by the Japanese Brain Bank Network for Neuroscience Research at Tokyo Metropolitan Institute for Geriatrics and Gerontology. Human tau was extracted from the postmortem brains of three individuals with pathologically confirmed PART according to a previously established protocol (Guo et al., 2016; He et al., 2018). Briefly, human brain tissue was homogenized in a lysis buffer using a Dounce homogenizer (Sigma, D8938). After centrifugation, the pellet was resuspended in the same buffer, and the supernatant was collected repeatedly. Sarkosyl was then added to the supernatant at a final concentration of 1%, incubated for 1 h, and centrifuged. The sarkosyl-insoluble pellet was collected as the fraction containing tau. After brief sonication and centrifugation, the final supernatant was used as the purified human brain-derived tau. All postmortem human brain tissue used in this study was histologically evaluated (Table 1). The use of human tissue was approved by the institutional review board of the RIKEN Center for Brain Science.

**Table 1.** PART cases employed in this study

Case #	Age	Sex	RNA integrity number	Senile plaque	Braak stage
1	79	F	3.63	1	3
2	88	M	6.53	1	3
3	93	F	7.5	1	3

### Intracerebral administration of purified human tau

For the tau-injected dKI (Tau-dKI) mice, human brain-derived tau (0.2 μg per mouse) was injected into the hippocampus and overlying cortex of the right hemisphere [bregma, −2.5 mm; lateral, 2 mm from midline; depth, −1.4 mm from skull (for the cortex) and −2.4 mm from skull (for the hippocampus)] in mice under anesthesia, using stereotaxic apparatus as described previously (Guo et al., 2016; He et al., 2018). In this study, 10–12-month-old homozygous dKI mice were used for tau inoculation. The mice were then maintained for 12–14 months to allow for tau propagation. Next, the 24-month-old Tau-dKI mice were perfused with phosphate-buffered saline (PBS), and the brains were quickly removed. The mouse brains were immediately stored at −80°C until their use in single-nucleus RNA-seq or were fixed with 4% PFA for immunohistochemical analyses.

### Immunohistochemistry

Frozen or paraffin-embedded sections were used. In the case of paraffin-embedded sections, the sections were deparaffinized and boiled for 5 min at 121°C in 10 mM sodium citrate buffer, pH 6.0, for antigen retrieval. After washing with PBS, all sections were then incubated with blocking buffer. Next, the sections were incubated with the following primary antibodies, diluted in blocking buffer: rabbit anti-ionized calcium-binding adapter molecule 1 (Iba1) antibody (FUJIFILM Wako Shibayagi, catalog #019-19741, RRID:AB_839504), chick anti-Iba1 antibody (Synaptic Systems, catalog #234 009, RRID:AB_2891282), rat anti-Dectin-1 antibody (InvivoGen, catalog #mabg-mdect, RRID:AB_2753143), goat anti-galectin-3 antibody (R&D Systems, catalog #AF1197, RRID:AB_2234687), rabbit anti-amyloid-β antibody (IBL-America, catalog #18584, RRID:AB_10705431), mouse anti-phosphorylated tau antibody (Fujirebio Europe NV, catalog #90206), mouse anti-phosphorylated tau antibody (Ser202, CP13), mouse anti-phosphorylated tau antibody (T231, RZ3), mouse anti-phosphorylated tau antibody (Ser396/Ser404, PHF-1), mouse anti-conformational epitope of tau antibody (including amino acids at 7–9 and 312–342, MC-1), mouse anti-tau (three-repeat isoform, RD3; Millipore, catalog #05-803, RRID:AB_310013), and mouse anti-tau (four-repeat isoform, RD4; Millipore, catalog #05-804, RRID:AB_310014). After washing with PBS again, the sections were incubated with the appropriate secondary antibodies. In the case of paraffin-embedded sections, fluorescent signals were enhanced using a TSA amplification kit (PerkinElmer Life Sciences). Digital data were captured using a NanoZoomer NDP system (Hamamatsu Photonics) or a TiE-A1R confocal laser scanning microscope (Nikon).

### Gallyas silver staining

Paraffin-embedded brain sections were deparaffinized, hydrated, incubated in 5% periodic acid for 3 min (mouse sections) or 5 min (human sections), and washed two times with distilled (d) H_2_O for 5 min. The sections were then incubated in silver iodide solution (150 ml of dH_2_O, 12 g sodium hydroxide, 30 g potassium iodide, 10.5 ml of 1% silver nitrate, and additional dH_2_O to a total volume of 300 ml) for 1 min. Next, the sections were placed into 0.5% acetic acid for 5 min twice before being rinsed in dH_2_O. Developer A (50 g anhydrous sodium carbonate in 1 L of dH_2_O), developer B (1.9 g ammonium nitrate, 2.0 g silver nitrate, and 10 g tungstosilicic acid in 1 L of dH_2_O), and developer C (1.9 g ammonium nitrate, 2.0 g silver nitrate, 10 g tungstosilicic acid, and 7.6 ml 37% formaldehyde in 1 L of dH_2_O) were prepared, and 200 ml of developer A, 100 ml of developer B, and 100 ml of developer C were then mixed together (in this order). Next, the sections were incubated in the developing solution until they turned pale brown/gray. The sections were then placed into 0.5% acetic acid for 5 min to stop the reaction before being dehydrated and coverslipped. The brain sections of female AD patients (ages 83 and 94) were used for the positive control (obtained from Tissue Solutions).

### Imaging data analysis

Iba1-positive (Iba1^+^) microglia were counted using Imaris software (v.9.8.2; Bitplane). Iba1^+^ nuclei were first visualized using the “colocalization” function. Next, “spots” were built using background subtraction to count these Iba1^+^ nuclei. The “estimated XY diameter” was set to 5 µm in all analyzed images. Any spots whose signals were on microglial processes or other cell parts were excluded. The “classification” function was used to distinguish Dectin-1^+^ and galectin-3^+^ microglia. For classification, the intensity mean of Dectin-1 was set at the *x*-axis, and the intensity mean of galectin-3 was set at the *y*-axis. Intense dot-like signals confined to small intracellular regions were considered autofluorescence. The same thresholds were applied to all images.

AT8-positive areas were quantified in the cortex, hippocampus, and corpus callosum using MetaMorph imaging software (Universal Imaging Corp.), as described previously (Saito et al., 2019).

### Single-nucleus RNA-seq

Cell nuclei were collected from the hippocampi of 24-month-old AD model mice according to the method used in a previous study (Habib et al., 2020). All procedures were performed on ice. Frozen tissue was transferred to a Dounce homogenizer and homogenized in EZ Lysis Buffer (Sigma-Aldrich, NUC101-1KT). The homogenate was left on ice for 5 min with a light shake every 1–2 min. The homogenate was centrifuged at 500 × *g* for 5 min, and the pellet was relysed in EZ Lysis Buffer. The sample was again centrifuged at 500 × *g* for 5 min, and the pellet was relysed in PBS containing 0.04% bovine serum albumin and 40 U/ml RNase inhibitor. After filtration through 40 μm Flowmi cell strainers (Sigma-Aldrich, BAH136800040-50EA), the crude suspension was collected into a 1.5 ml tube. After the nuclei were stained with 1 μg/ml 4′,6-diamidino-2-phenylindole, cell-sorting–based purification was performed using a FACSAria Ⅱ (Becton, Dickinson and Company). The purified nucleus suspension was then adjusted to a concentration of 700–1,200 nuclei/ml using a Countess 2 Cell Counter (Thermo Fisher Scientific) to load 10,000 cells/reaction into the 10x Genomics Chromium controller for single-cell RNA-seq. A Chromium Next GEM Single Cell 3′ Kit v3.1 was used for reverse transcription, cDNA ampliﬁcation, and library construction following the detailed protocol provided by 10x Genomics. Libraries were quantiﬁed using a Qubit 2.0 Fluorometer (Thermo Fisher Scientific); quality was confirmed using a 2100 Bioanalyzer with High Sensitivity DNA kit (Agilent Technologies). Sequencing was performed in paired-end mode (read1, 28 bp; read2, 90 bp) using a NovaSeq 6000 sequencer (Illumina).

### Single-nucleus RNA-seq sequence data analysis

Sequence data were analyzed using Seurat (https://satijalab.org/seurat/) version 5.0.2 (Hao et al., 2024) implemented in R version 4.3.2. First, filtered gene expression matrices were converted into Seurat objects using the CreateSeuratObject function. We selected cells with >400 genes expressed and a mitochondrial gene ratio <1%. Possible doublet populations were identified by DoubletFinder (McGinnis et al., 2019), with an estimated doublet ratio of 20%, and were removed for the following analyses. The average numbers of reads and genes per cell were 11,218 and 3,103, respectively. Data from each sequence were normalized using the SCTransform function (Hafemeister and Satija, 2019) before being integrated with the FindIntegrationAnchors and IntegrateData functions (Stuart et al., 2019). The principal components were calculated from the variable genes of the integrated Seurat object. Clusters were identified using FindNeighbors and FindClusters, with a resolution of 0.05. We generated two-dimensional projections using the Uniform Manifold Approximation and Projection (UMAP) algorithm based on the top 30 principal components. Cluster annotation was conducted by referring to the expression patterns of existing cell-type markers. Additional clustering analyses were then performed on *Hexb*^+^ microglia, with a resolution of 0.2. Microglia clusters expressing other cell-type markers, such as *St18* (for oligodendrocytes) and *Rbfox3* (for neurons), were judged as doublets and eliminated from the subsequent analyses. Marker genes for each subcluster of microglia were searched using the FindAllMarkers function. Differential expression analysis was performed according to a previous study (Zhan et al., 2020) using the FindMarkers function with default Wilcoxon rank-sum test and log fold-change threshold set at 0.5. Enrichment analyses were performed in Metascape (Zhou et al., 2019) using the identified marker genes. The single-cell RNA-seq and spatial transcriptomic datasets generated in the present study have been deposited in the Gene Expression Omnibus (GEO) repository under accession number GSE270389.

### Pseudotime analyses

Pseudotime analyses were performed with Monocle 3 (Cao et al., 2019) using the final microglial dataset generated by Seurat. The cell_data_set object was created using the new_cell_data_set function. The required information for running the function (i.e., gene expression matrix, metadata of cells, and metadata of genes) was transferred from the Seurat object. We also used the UMAP embeddings in the Seurat object instead of running the following Monocle functions: preprocess_cds, reduce_dimension, and cluster_cells. The trajectory on the UMAP projections was calculated using the learn_graph function. The homeostatic population was selected for the roots of the trajectory, and the cells were arranged in order using the orders_cell function. The plot_cells function was used for visualization.

### Reanalyses of public datasets

Publicly available single-cell RNA-seq datasets of AD mouse models were used to validate and strengthen our results. Sequence data were analyzed using Seurat on R version 4.3.2. *App^NL-G-F^* datasets (GSE127893; Frigerio et al., 2019) were used to calculate the similarity score and explore age-dependent effects on the identified cluster markers. An aged mouse dataset (GSE166548; Safaiyan et al., 2021) was used to calculate the similarity score to white matter-associated microglia (WAMs). 5XFAD datasets (GSE140510; Zhou et al., 2020) were used to explore TREM2-deficient effects. P301S datasets (GSE198014; C. Wang et al., 2022) were examined to determine whether the identified marker genes could also be detected in the tauopathy models. The gene expression matrix was converted to a Seurat object and normalized using the NormalizeData function. Two thousand variable genes were identified using the FindVariableFeatures function. Clusters were then explored using the top 30 principal components and a resolution of 0.1. For the *App^NL-G-F^* datasets, subclustering analyses of microglial populations were conducted with the top 16 principal components and a resolution of 0.1; for the 5XFAD datasets, analyses were conducted with the top 20 principal components and a resolution of 0.2.

### Spatial transcriptomics

For spatial whole transcriptome profiling, we used the Visium Spatial Gene Expression platform (10x Genomics) according to the manufacturer's instructions. Subsequently, libraries were sequenced on a NovaSeq 6000 (Illumina) in paired-end mode (read1, 28 bp; read2, 120 bp). The resulting raw reads were processed using Space Ranger 1.0.0 (10x Genomics).

### Spatial transcriptomics sequence data analysis

All analyses were conducted using Seurat version 5.0.2 implemented in R version 4.3.2. Sequence data were transformed into Seurat objects using the Load10x_Spatial function before being normalized using the SCTransform function. The average numbers of reads and genes per spot were 27,330 and 5,753, respectively. Principal components were calculated from the variable genes using the RunPCA function. The top 30 principal components were used for the UMAP plot. Cell clusters were identified using the FindNeighbors and FindClusters functions with a resolution of 0.1. The integration of spatial transcriptomic data with single-cell RNA-seq data was performed using the TransferData function after finding the anchors in the sequence data using the FindIntegrationAnchors function.

### RNAscope

All procedures were performed using a Manual Fluorescent Multiplex kit v2 (Advanced Cell Diagnostics) according to the manufacturer's protocol. After deparaffinization and antigen retrieval, mouse brain sections were incubated at 40°C for 2 h with the following probes: Mm-Ctnna3 (516621), Mm-Cxcl16 (466681), Mm-Dkk2 (404841), and Mm-Cst7 (498711). Signal amplification was performed after several washing steps. The sections were then incubated with detection buffer until the signal reached the appropriate level under microscopic observance. Data were captured using a FluoView FV3000 (Olympus).

### Statistical analyses

Data were first analyzed for normal distribution and equal variance. Normally distributed data were analyzed using a two-tailed Student's *t* test or Welch's *t* test. If the data did not pass normality testing, the Mann–Whitney *U* test was used. *p *< 0.05 was considered significant. All analyses were conducted using R version 4.3.2.

## Results

### Generation of a humanized AD mouse model with tau propagation and accumulation

Previous studies have established a protocol for the intracerebral injection of human brain-derived tau into amyloid model mice to recapitulate the spreading process of pathological tau in vivo (He et al., 2018; Vergara et al., 2019). AD model mice exhibit the robust propagation and accumulation of endogenous murine tau not only in the injected side but also in the contralateral side of the brain (He et al., 2018). In the present study, we performed two modifications of the established protocol to capture tau-related alterations at the single-cell level. First, we used the recently established *App^NL-G-F^*/*MAPT* dKI mouse (Hashimoto et al., 2019; Saito et al., 2019) as an ideal in vivo platform for studying AD tauopathy. In the *App^NL-G-F^* model, amyloid-β is humanized with three pathogenic AD mutations, and the mice show cortical amyloid deposition from just 2 months of age (Saito et al., 2014). In the dKI model, the *Mapt* locus in *App^NL-G-F^* mice is further humanized to recapitulate the three- and four-repeat tau splicing forms. Expression of three- and four-repeat human tau is important because both isoforms accumulate in tau aggregates of AD and PART patients (Gotz et al., 2019). Second, we extracted and purified human tau from the temporal cortex of aged individuals with PART (Crary et al., 2014; Ferrer et al., 2020). Given the presence of amyloid-β fibrils in both aqueous extracts and insoluble fractions from AD brains (Stern et al., 2023), it is difficult to purify tau only from AD brains. Unlike AD, PART cases show age-related tau accumulation only, either without or with minimal amyloid pathology and brain atrophy. We therefore hypothesized that it would be possible to collect purified human tau from PART brains, thus minimizing the potential pathological contaminants of tau from the AD brain. Collectively, we expected that this new mouse model would exhibit the progression status of tau pathology in a more precise manner compared with previous models.

**Figure 1.**
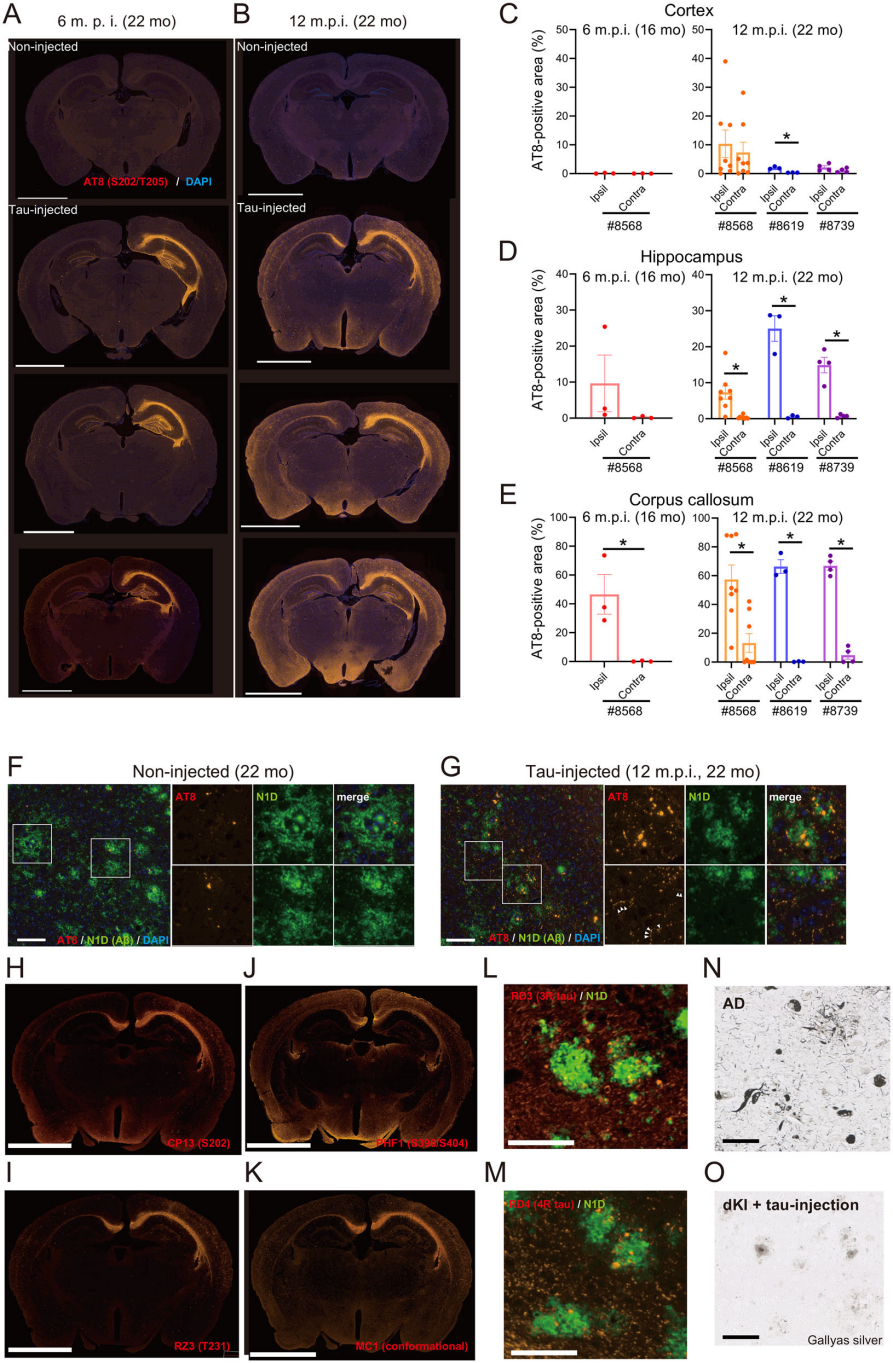
Generation of a humanized AD mouse model with tau propagation and accumulation. ***A***, ***B***, Immunohistochemical analysis of phosphorylated tau in dKI mice at 6 (***A***) and 12 (***B***) months after PART tau injection. The AT8 (S202/T205) antibody was used to visualize phosphorylated tau (red). Scale bars, 2.5 mm. ***C–E***, The area of AT8 signal was quantified by image analysis. PART tau was prepared from three subjects (#8568, #8619, #8739). Each dot represents an experimental point [*n* = 3 (6 m.p.i. #8568), *n* = 8 (12 m.p.i. #8568), *n* = 3 (12 m.p.i. #8619), and *n* = 4 (12 m.p.i. #8739), where each *n* reflects one mouse; *p < 0.05, *t* test. ***F***, ***G***, High-magnification images of phosphorylated tau (red) and amyloid plaques (green) in noninjected dKI (***C***) and Tau-dKI (***D***) mice. The N1D antibody (Saido et al., 1995) was used to visualize amyloid plaques. Scale bars, 100 μm. ***H–K***, Immunohistochemical analyses using various tau antibodies against phosphorylated tau (***H***, CP13, S202; ***I***, RZ3, T231; ***J***, PHF1, S396/S404) or tau with altered structure (***K***, MC1). Scale bars, 2.5 mm. ***L***, ***M***, Immunostaining of three-repeat (***L***) and four-repeat (***M***) tau using specific antibodies (red) against each isoform and amyloid plaques (green). Note that both three- and four-repeat tau accumulated in the NP tau pathology. Scale bars represent 100 μm. ***N***, ***O***, Gallyas silver staining in the AD brain (***K***) and in Tau-dKI mice (***L***). Scale bars, 100 μm.

Six months after human PART tau injection, phosphorylated tau signal was widely detected in the Tau-dKI hippocampus and corpus callosum (Fig. 1*A*,*C–E*). Twelve months after human tau injection, time-dependent tau propagation was observed in a broad range of brain regions, including the ipsilateral cortex and contralateral hippocampus (Fig. 1*B–E*). We also detected less abundant phosphorylated tau in the ipsilateral hippocampus at 12 m.p.i. (Fig. 1*B*). The tau generated in the hippocampus at 6 m.p.i. might have been gradually transported to other regions over time. A previous study has also reported that AT8 signals were reduced in the tau-injected hippocampus at later stages (Guo et al., 2016). These results have supported the hypothesis that PART tau may be the first seed of tau pathology in AD. Consistent with a previous study, AT8^+^ phosphorylated tau signal was increased around amyloid-β (NP tau) in the Tau-dKI mice compared with the noninjected control mice (Fig. 1*F*,*G*). In addition, we observed a significant increase of neuropil-thread–like punctate signals of phosphorylated tau. Immunohistochemical analyses using various tau antibodies revealed that the phosphorylated tau with an altered structure was contained and accumulated in the model mouse brain (Fig. 1*H–K*). Furthermore, both three- and four-repeat tau was accumulated within the NP tau (Fig. 1*L*,*M*). However, unlike in AD human patients and tau transgenic mice, we did not find apparent neurofibrillary-tangle–like structures after Gallyas silver staining in Tau-dKI mice (Fig. 1*N*,*O*), suggesting that this new model recapitulates a relatively early stage of the disease, with pretangle formations. Together, these results indicate the successful generation of a novel AD model mouse with humanized amyloid-β and tau accumulation in the brain, without any transgene overexpression.

### Increased DAM in the Tau-dKI mouse hippocampus

**Figure 2.**
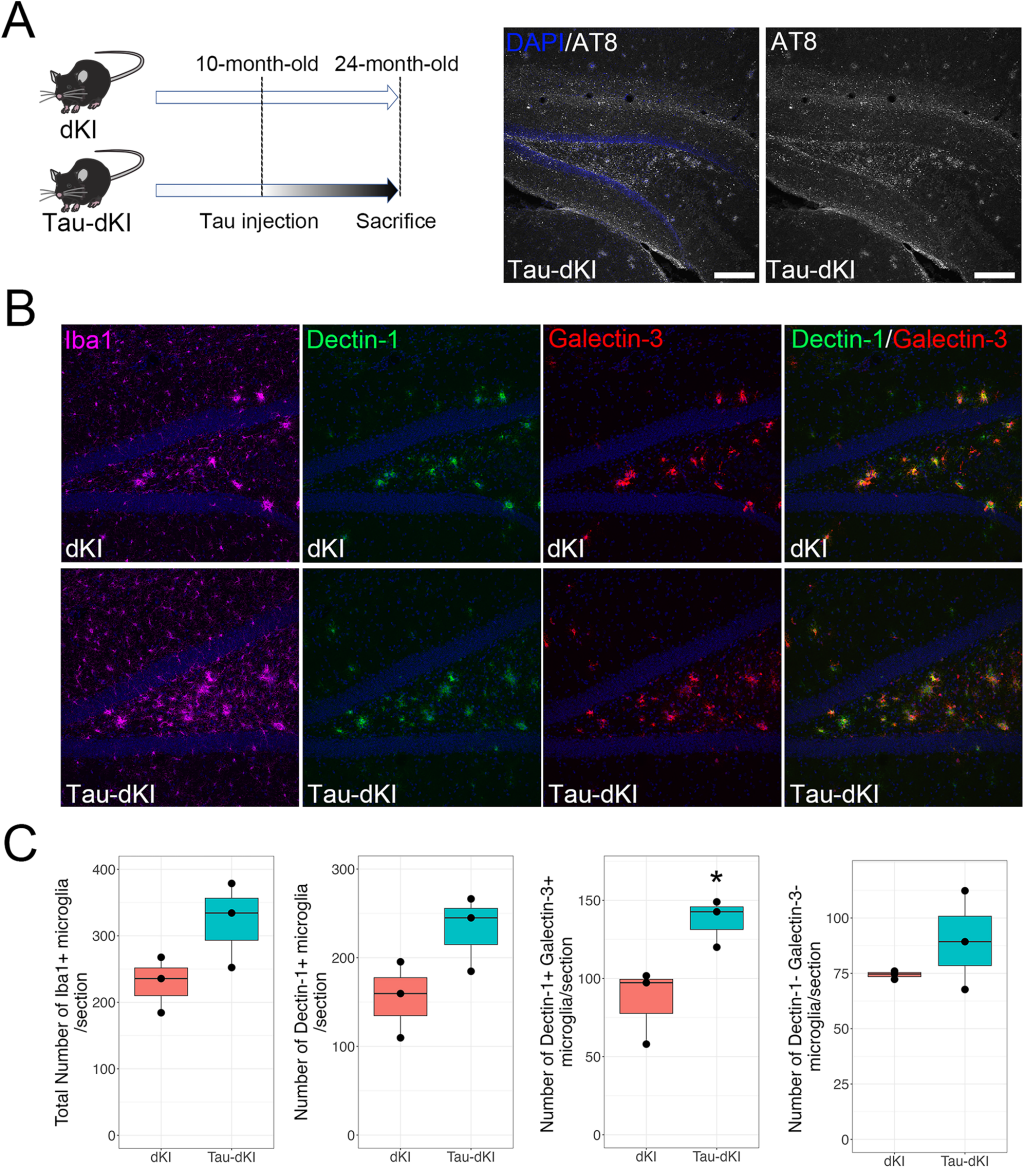
Increased Dectin-1^+^ microglia are associated with tau accumulation in the hippocampus. ***A***, Left, Schematic diagram showing the timings of tau injection and brain sampling. Right, AT8^+^ hyperphosphorylated tau accumulation (AT8) in the injected side of the hippocampus. Cell nuclei were visualized with 4′,6-diamidino-2-phenylindole (DAPI; blue). ***B***, Confocal images of hippocampal sections stained with antibodies against Iba1 (purple), Dectin-1 (green), and galectin-3 (red). Dectin-1 and galectin-3 colocalization is shown as white. Cell nuclei were visualized with DAPI (blue). Images are taken from the dentate gyrus region. The top panels are representative images from dKI mice, and the bottom panels are representative images from Tau-dKI mice. Scale bars, 100 μm. ***C***, Quantitative analyses of microglia in 24-month-old dKI and Tau-dKI mice. The numbers of Iba1^+^ microglia, Dectin-1^+^ microglia, Dectin-1/galectin-3 double-positive microglia, and Dectin-1/galectin-3 double-negative microglia are presented as boxplots (each black dot represents an experimental point; *n* = 3 mice per group; *p < 0.05, *t* test).

We first histologically evaluated the differences in microglia between 24-month-old dKI and Tau-dKI mice. The Tau-dKI mice received an injection of PART tau into the ipsilateral hippocampus and overlying cortex at 10–12 months of age. At 24 months of age, phosphorylated tau signals were widely distributed in the hippocampus, especially in the polymorphic cell layer of the dentate gyrus, in the Tau-dKI mice (Fig. 2*A*). Although quantitative analyses indicated an apparent increase in Iba1^+^ microglial number in the Tau-dKI hippocampus compared with dKI mice, this difference was not significant (Fig. 2*B*,*C*). Furthermore, many microglia had altered morphology and multiple microglia appeared to be clustered, especially in the polymorphic cell layer (Fig. 2*B*,*C*). Immunohistochemistry revealed that the number of Dectin-1^+^ (a marker of DAM) microglia was increased in the hippocampus of Tau-dKI mice compared with dKI mice (Fig. 2*B*,*C*). These results indicate that, similar to amyloid deposition, tau accumulation also increases the numbers of activated microglia in the brain.

We also performed immunochemistry for another DAM marker, galectin-3 (also called Mac2). Galectin-3 is highly accumulated in both AD model (Boza-Serrano et al., 2019) and tau transgenic (Siew et al., 2024) mouse brains. Importantly, a loss of galectin-3 reduces amyloid accumulation and AT8^+^ regions in AD model mice (Siew et al., 2024), suggesting that galectin-3 may play an important role in AD pathogenesis. Galectin-3 also accumulates in the brains of patients with AD and frontotemporal lobar degeneration (Boza-Serrano et al., 2019; Siew et al., 2024). In the present study, similar to Dectin-1, intense galectin-3 signals were detected in the hippocampus. A substantial number of microglia were also detected as Dectin-1/galectin-3 double-positive cells in both the dKI and Tau-dKI mice (Fig. 2*B*,*C*). Similar to the increase in Dectin-1^+^ cells, the number of double-positive microglia was significantly increased in Tau-dKI mice (*p* = 0.035). In contrast, the number of nonactivated homeostatic microglia (negative for both Dectin-1 and galectin-3) did not change with tau accumulation (Fig. 2*C*).

### Multiple DAM subpopulations in the 24-month-old AD model mice

**Figure 3.**
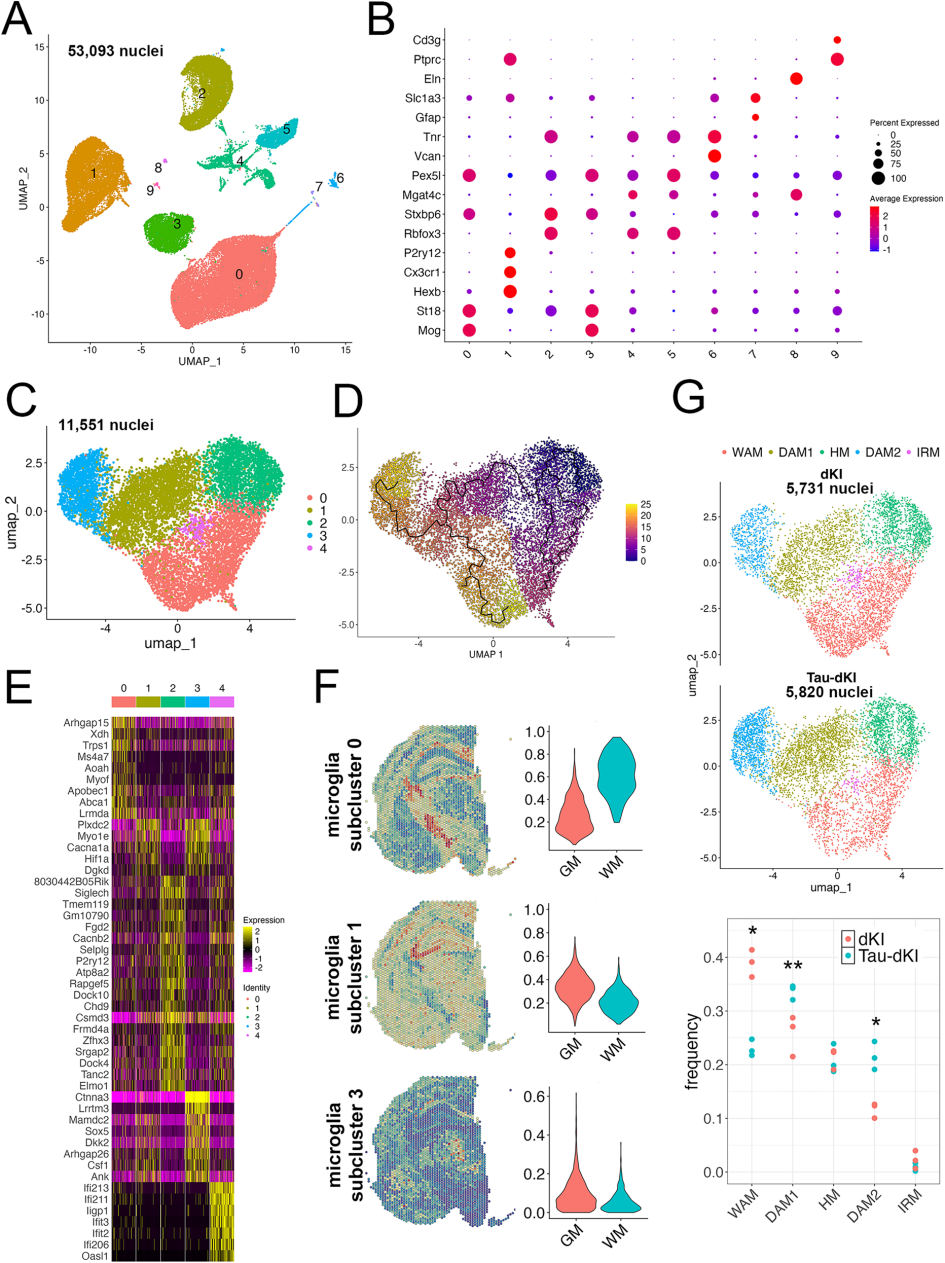
Alteration of microglial states in the Tau-dKI hippocampus. ***A***, UMAP plot based on the gene expression of 53,093 single nuclei. Ten clusters (0–9) were discriminated. Extended Data Figure 3-1 shows the expression of selected marker genes of the clusters. ***B***, Dot plot showing average gene expression levels and the percentages of cells expressing marker genes across all clusters. *Hexb*, *Cx3cr1*, *P2ry12*, and *Tmem119* were used as marker genes for microglia. Identified marker genes of all clusters are added as Extended Data Table 3-1. ***C***, UMAP plot of the 11,551 single microglial nuclei that passed quality control after the removal of other border-related macrophages. Five microglial subclusters (0–4) were detected in both dKI (*n* = 3) and Tau-dKI (*n* = 3) mice. Extended Data Figure 3-2 includes information of all Hexb+ population subtracted from the whole dataset. Extended Data Figure 3-3 shows similarity scores for the known states in each microglial subcluster. ***D***, UMAP projection plot of all microglial nuclei with color mapping to the transcriptional pseudotime, calculated using Monocle 3. ***E***, Gene expression heatmap for the microglial clusters. The genes shown on the left are the top gene markers for each microglial subcluster. See Extended Data Figure 3-4 for Ctnna3 expression in our Tau-dKI model and other AD models, Extended Data Figure 3-5 for histological distribution of Ctnna3 mRNA in aged AppNL-G-F brain. ***F***, Integrative analyses of spatial transcriptomics using a single-nucleus RNA-seq dataset. The likelihood of the presence of a microglial subpopulation at each spot on the injected side of a Tau-dKI brain section was estimated from single-nucleus RNA-seq data. Subcluster 0 was shown to predominantly localize to white matter (WM). The remaining two subclusters were present in gray matter (GM) at least as much as in WM. Extended Data Figure 3-6 includes basic information of the spatial transcriptomics. ***G***, UMAP plot of microglia in dKI (5,731 nuclei) and Tau-dKI (5,820 nuclei) mice. Dot plot showing the frequencies of nuclei per cluster in dKI and Tau-dKI mice (each colored dot represents the cluster frequency from an experimental sample; *n* = 3 mice per group; *p < 0.05, *t* test, **p < 0.01, *t* test). The names of microglial subclusters (0–4) were changed to the previously defined states: white matter-associated microglia (WAM), intermediate disease-associated microglia (DAM1), homeostatic microglia (HM), disease-associated microglia (DAM2), and interferon response microglia (IRM). Differential expression analyses were performed between Tau-dKI and dKI (Extended Data Fig. 3-7).

To capture the alterations of detailed cellular states during tau accumulation and propagation after amyloid-β accumulation, we further performed single-nucleus RNA-seq on the hippocampi of 24-month-old dKI and Tau-dKI mice. Generated sequence data from 53,093 nuclei were integrated for the analyses. The ratio of expressed mitochondrial genes was extremely low, and the number of genes was almost the same in all analyzed samples, indicating that possible cytoplasmic contamination and/or technical variations between samples were minimal in our cell-sorting–based procedure (Extended Data Fig. 3-1). When we plotted the cellular features in two dimensions of the UMAP, 10 clusters were discriminated (Fig. 3*A*). Based on marker gene expression, *St18*^+^ oligodendrocytes (clusters 0 and 3), *Rbfox3*^+^ neurons (clusters 2, 4, and 5), *Hexb*^+^ microglia (cluster 1), and *Vcan*^+^ oligodendrocyte precursor cells (cluster 6) were the main cell types (Fig. 3*B*, Extended Data Fig. 3-1). Unexpectedly, there were relatively few *Slc1a3*^+^ astrocytes in our model. There were no clear differences in the frequencies of each cell population between the dKI and Tau-dKI mice (Extended Data Fig. 3-1). Marker genes of all clusters are listed in Extended Data Table 3-1.

10.1523/ENEURO.0260-24.2024.f3-1 Fig 3-1 Basic information about the whole single-nucleus RNA-seq dataset **A** Violin plot showing the distributions of the number of detected genes, number of detected transcripts, and percentage of mitochondrial genes in each group. **B** Violin plot showing the distribution of the number of detected genes in each sample. **C** UMAP plots colored by the normalized expression levels of selected marker genes: *Rbfox3* for neurons, *St18* for oligodendrocytes, *Hexb* for microglia, *Vcan* for oligodendrocyte precursor cells, *Slc1a3* for astrocytes, and *Gfap* for activated astrocytes. **D** Dot plot showing the frequencies of nuclei per cluster in dKI (red) and Tau-dKI (blue) mice. Download Fig 3-1, TIF file.

10.1523/ENEURO.0260-24.2024.t3-1 Table 3-1 Identified cell cluster markers. Download Table 3-1, XLS file.

10.1523/ENEURO.0260-24.2024.f3-2 Fig 3-2 Characteristics of the *Hexb*^+^ population subtracted from the whole dataset **A** UMAP plot of the *Hexb*^+^ cluster (cluster 1 in Fig. 3) based on the recalculated scores. **B** Dot plot showing the average gene expression levels and the percentages of cells expressing marker genes across all clusters. Clusters 4 and 6 were likely doublet clusters because of their high expression of the non-microglial markers *St18* and *Rbfox3*, respectively. Cluster 5 was evaluated to be a central nervous system border-associated macrophage because of the dominant expression of *Mrc1*. **C** Violin plots showing the distributions of the detected genes across the eight clusters. The average numbers of detected genes were relatively high in both clusters 4 (4,323 genes) and 6 (3,406 genes) compared with clusters 0–3 (2,422 genes). We also determined cluster 7 to be a homophilic doublet cluster because of the number of detected genes (3,628 genes). **D** UMAP plots, colored by the normalized expression levels of selected marker genes: *Hexb* for microglia, *Mrc1* for central nervous system border-associated macrophages, *Rbfox3* for neurons, and *St18* for oligodendrocytes. Download Fig 3-2, TIF file.

10.1523/ENEURO.0260-24.2024.f3-3 Fig 3-3 Characteristics of microglial subclusters **A** Similarity scores for the known microglial states in each cluster. We used sequence data to calculate similarity scores for DAM, homeostatic microglia (HM), interferon response microglia (IRM; all from accession number NCBI: GSE127893), and WAM (from accession number NCBI: GSE166548). **B** Gene Ontology terms associated with the genes enriched in microglial clusters 0 (upper) and 3 (lower). Enrichment analyses were performed using Metascape. Download Fig 3-3, TIF file.

10.1523/ENEURO.0260-24.2024.f3-4 Fig 3-4 *Ctnna3* expression in our Tau-dKI model and other AD models **A** (Left) UMAP plots colored by the normalized expression levels of selected genes. *Hexb* is a marker for microglia and *Cst7* is a marker for DAM. (Right) Violin plots showing *Cst7*, *Ctnna3*, and *Dkk2* expression in 3-, 6-, 12-, and 21-month-old *App^NL-G-F^* mice. Sequence data from accession number NCBI: GSE127893. **B** (Left) UMAP plots colored by the normalized expression levels of selected genes. (Right) Violin plots showing *Cst7*, *Ctnna3*, and *Dkk2* expression. Similar to known DAM markers, *Ctnna3* expression was detected in 5XFAD microglia but not TREM2-deficient 5XFAD microglia. Sequence data from accession number NCBI: GSE140510. **C** (Left) UMAP plots colored according to the normalized expression levels of selected genes. (Right) Violin plots showing *Cst7*, *Ctnna3*, and *Dkk2* expression. In contrast to known DAM markers, *Ctnna3* expression was barely detected in P301S microglia. Sequence data from NCBI accession number: GSE198014. **D** (Left) UMAP plots colored according to the normalized expression levels of selected genes in Tau-dKI microglia. (Right) Violin plots showing *Cst7*, *Ctnna3*, and *Dkk2* expression. Download Fig 3-4, TIF file.

10.1523/ENEURO.0260-24.2024.f3-5 Fig 3-5 Histological distribution of *Ctnna3* mRNA in aged *App^NL-G-F^* brain Representative RNAscope images of *in situ* hybridization and immunohistochemical staining on 24-month-old *App^NL-G-F^* brain sections, which were used to evaluate the spatial distribution of selected marker genes. *Ctnna3* mRNA was partially colocalized with microglia surrounding amyloid-β. Scale bar: 50 μm. Download Fig 3-5, TIF file.

10.1523/ENEURO.0260-24.2024.f3-6 Fig 3-6 Spatial transcriptomics and integrative analyses with single-nucleus RNA-seq **A** Schematic diagram showing the experimental procedures of spatial transcriptomics. **B** UMAP plot of the 11,551 spots. **C** The six discriminated clusters corresponded to anatomical brain regions. **D** Integrative analyses of the spatial transcriptomics in a Tau-dKI brain section (injected side). The likelihood of the presence of each of the six clusters (0 to 5) at each spot on the section was estimated from single-nucleus RNA-seq data. *St18* and *Mog* high oligodendrocyte clusters (0 and 3), *Hexb*^+^ microglia cluster (1), and *Rbfox3*^+^ neuronal clusters (2, 4, and 5). Download Fig 3-6, TIF file.

10.1523/ENEURO.0260-24.2024.f3-7 Fig 3-7 Differential expression analyses between Tau-dKI and dKI **A** Volcano plot showing differentially expressed genes identified by comparison of Tau-dKI vs. dKI. **B** UMAP plots colored according to the normalized expression levels of selected genes. **C** Violin plots showing the distributions of the detected genes in each group. **D** Gene Ontology terms associated with the enriched downregulated (upper) and upregulated (lower) genes in Tau-dKI. Enrichment analyses were performed using Metascape. Download Fig 3-7, TIF file.

The *Hexb*^+^ cell population was then extracted for further analysis. After removing doublet clusters and central nervous system border-associated macrophages (Extended Data Fig. 2), five microglial subpopulations were detected (Fig. 3*C*). We used an existing single-cell RNA-seq dataset from microglia (Frigerio et al., 2019) to assess which subcluster was DAM. Unexpectedly, multiple subclusters were estimated as DAM: the evaluation based on the existing dataset estimated that subclusters 0, 1, and 3 showed DAM signatures at high levels. In contrast, subcluster 2 was evaluated to be homeostatic microglia (Extended Data Fig. 3-3). Subcluster 4 appeared to be another microglial nonhomeostatic state, known as interferon response microglia (Frigerio et al., 2019). Using subcluster 2 as a starting point, the pseudotime analysis revealed that the transition of microglial states partly diverged over time and distinguished two distinct subclusters—subclusters 0 and 3—at the endpoints (Fig. 3*D*). Notably, the enriched Gene Ontology terms were different between the two subclusters (Extended Data Fig. 3-3). These results suggest that the identified microglia contain multiple DAM-like populations with different properties.

Next, we analyzed differentially expressed genes to extract unique markers in the identified microglial subclusters. Subcluster 1 microglia highly expressed known DAM markers, including *Myo1e* and *Hif1a*, as cluster markers (Fig. 3*E*). In addition to the DAM markers detected in subcluster 1, subcluster 3 expressed several unique marker genes (Fig. 3*E*). Of these, *Ctnna3*, which encodes catenin alpha-3 (Weng et al., 2022), was of particular interest not only because the gene was exclusively expressed in subcluster 3 but also because it has been reported in association with AD risk (Miyashita et al., 2007; Ghani et al., 2013). However, because it was unclear whether *Ctnna3* was really a DAM marker, we further explored the expression pattern of *Ctnna3* in microglia from AD model mice using several existing single-cell RNA-seq datasets of microglia. *Ctnna3* expression was partially detected in *Cst7*^+^ DAM in *App^NL-G-F^* mice, a knock-in mouse model (Extended Data Fig. 3-4). Although *Ctnna3* expression was quite rare at 3 months of age, it was substantially expressed from 6 months of age, when amyloid plaques were highly accumulated in the brain (Saito et al., 2014; Extended Data Fig. 3-4). Furthermore, similar to the *App^NL-G-F^* dataset, *Ctnna3*^+^ microglia clearly appeared in a subset of the *Cst7*^+^ population in 5XFAD mice, an overexpressing mouse model (Zhou et al., 2020; Extended Data Fig. 3-4). However, this microglial subpopulation was completely depleted in 5XFAD mice with TREM2 deficiency (Extended Data Fig. 3-4). In contrast to the amyloid-β models, the Cst7^+^ population was very limited in the P301S tauopathy mouse model, and we could barely detect *Ctnna3^+^* microglia in this model (Extended Data Fig. 3-4).

We also evaluated the expression of *Dkk2*, another subcluster 3 marker, in the existing datasets. Although *Dkk2*, which encodes an endogenous Wnt inhibitor, was not identified in the original DAM paper (Keren-Shaul et al., 2017), it has been characterized as a DAM marker in subsequent studies (Frigerio et al., 2019; Aghaizu et al., 2023). Our reanalyses of *Dkk2* revealed that it was sparsely detected in DAM in a TREM2-dependent manner (Extended Data Fig. 3-4). Finally, we performed RNAscope to explore the spatial relationship between the representative subcluster 3 markers and AD pathology. Similar to *Cst7*, *Ctnna3*^+^ and *Dkk2*^+^ signals were detected in microglia surrounding amyloid-β (Extended Data Fig. 3-5). These results suggest that *Ctnna3* was partially expressed in DAM, as noted in previous bulk RNA-seq data (Meilandt et al., 2020).

### Tau accumulation increases DAM at the expense of white matter-associated subpopulations

Single-cell RNA-seq is a valuable tool for capturing the detailed cellular states of any cell type of interest. However, because information regarding cellular distribution in tissue is lost during the cell isolation processes, it is often difficult to appropriately interpret the identified clusters. As indicated by previous studies (S. H. Lee et al., 2021; Kim et al., 2022), DAM can often be discriminated into multiple subpopulations, possibly reflecting different samples and experimental conditions. The situation has been further complicated by the recent identification of DAM-like microglia in the normal aged brain without amyloid-β accumulation (Safaiyan et al., 2021). The age-related nonhomeostatic counterpart of DAM, WAM, was originally identified in the white matter of the aged mouse brain (Safaiyan et al., 2021). Although WAMs have also been detected in sequence datasets from AD model mouse brains, they have not been well characterized in brain sections from AD model mice because there is a lack of specific histological markers that distinguish WAM from DAM.

To obtain additional characteristics of the three identified microglial subpopulations with DAM signatures in the present study, we performed spatial transcriptomics on brain sections from 24-month-old Tau-dKI mice. Spatial transcriptomics can evaluate the expression of thousands of genes, along with spatial information, in a tissue of interest (Stahl et al., 2016; Chen et al., 2020). Although the technology alone cannot provide cellular-level annotation because of its low spatial resolution, a recently developed integrative method has allowed spatial information to be transferred to the cell populations identified in single-cell RNA-seq (Stuart et al., 2019). In the present study, cellular mRNA in the injected side of the Tau-dKI brain was captured on thousands of spots (each 55 μm in diameter) using oligonucleotides with spatial barcodes (Extended Data Fig. 3-6). In the resulting UMAP plot, six populations were discriminated, which were well matched to the anatomical brain regions (Extended Data Fig. 3-6). Subsequent integrative analyses clearly showed differences in the spatial distributions of major clusters from our single-nucleus RNA-seq (Extended Data Fig. 3-6). Importantly, the integrative analyses also revealed differences in the spatial distributions of microglial subclusters (Fig. 3); subcluster 0 was enriched in the white matter, whereas the remaining two subclusters (1 and 3) were located more in the gray matter than in the white matter. Collectively, we linked the three identified DAM-like clusters and their states. We assessed that subcluster 0 was WAM (Safaiyan et al., 2021; Kaya et al., 2022) based on its spatial distribution and high levels of DAM signatures. Consistent with our assessment, subcluster 0 uniquely expressed representative WAM markers, such as *Abca1* (Fig. 3*E*), and showed a high similarity score to WAM (Extended Data Fig. 3-3). Subcluster 1 was determined to be an intermediate DAM (DAM1) population based on its expression of known DAM markers and its location on the pseudotime axis. Subcluster 3 was evaluated to be DAM (DAM2) because of its strong expression of DAM genes as well as its location at the end of the pseudotime axis.

When we compared the frequencies of the identified microglial states, there were clear differences between dKI and Tau-dKI mice. The frequencies of DAM1 and DAM2 were significantly higher in Tau-dKI mice than in dKI mice, whereas that of WAM was much lower (Fig. 3*G*). To discriminate biological effects from technical variations, we repeated the single-nucleus RNA-seq with one dKI sample and one Tau-dKI sample three times with independent biological samples and obtained similar results. In contrast, the frequencies of homeostatic microglia and interferon response microglia did not change between the groups (Fig. 3*G*). We also searched for differentially expressed genes between Tau-dKI and dKI. Consistent with the microglial state frequency results, we found a number of genes with altered expression in Tau-dKI (Extended Data Fig. 3-7). Several genes that showed dominant expression in microglial subcluster 0, such as *Abca1* and *Apobec1*, were significantly downregulated in the Tau-dKI samples. In contrast, *Ctnna3*, a representative marker of microglial subcluster 3, was upregulated in the Tau-dKI samples. Using the identified gene lists, we performed enrichment analyses and found that the enriched Gene Ontology terms were clearly different between the two samples (Extended Data Fig. 3-7).

## Discussion

In the present study, we generated a novel AD model mouse, Tau-dKI, which showed robust Aβ and humanized tau accumulation without any transgene overexpression. At 24 months of age, Dectin-1^+^ and/or galectin-3^+^ DAM populations were increased in the polymorphic cell layer of the dentate gyrus, where tau intensively accumulated. Single-nucleus RNA-seq revealed that the frequencies of the three microglial subpopulations with DAM signatures differed between the uninjected dKI and Tau-dKI mouse hippocampus. Combined with findings from existing single-cell RNA-seq datasets and integrative analyses using spatial transcriptomics, we have provided the first evidence that human tau accumulation increases DAM and decreases WAM.

The advent of single-cell technology has led to the identification of DAM in AD model mice as well as AD patients (Keren-Shaul et al., 2017; Frigerio et al., 2019; Mathys et al., 2019; Zhou et al., 2020). The TREM2-deficient AD model, in which DAM are almost depleted, shows exacerbated amyloid pathology and neuritic dystrophy in the brain (Y. M. Wang et al., 2015; Yuan et al., 2016). Furthermore, TREM2-overexpressing mice have reduced amyloid deposition (C. Y. D. Lee et al., 2018). Similarly, AD patients with TREM2 mutations reportedly have fewer microglia around amyloid-β, which supports the findings from mice (Yuan et al., 2016). The suggested protective role of DAM against amyloid-β accumulation has led to the idea that actively increasing DAM in the brain may slow AD progression. Injections of TREM2 agonistic antibodies, which boost the numbers of DAM in the brain, have succeeded in inhibiting amyloid pathology in mice, and related phase 2 clinical trials are currently being conducted to develop new drugs for AD (S. T. Wang et al., 2020). However, although DAM seem to be protective against amyloid-β, they likely have an opposite effect on tau pathology; neurodegeneration caused by tau accumulation is markedly improved in APOE- and TREM2-deficient tau transgenic models (Leyns et al., 2017; Shi et al., 2017). This is also true for TREM2 agonistic antibodies; the intraperitoneal injection of TREM2 agonistic antibodies worsens tau pathology in an AD mouse model (Jain et al., 2022). Thus, a better understanding of how DAM responds to amyloid-β and tau accumulation, respectively, will not only help to determine when and how TREM2 agonistic antibodies should be administered to AD patients but will also be important for further elucidating the pathogenic mechanisms of AD.

In the present study, we have created a novel mouse model that combines genetic alterations and the intracranial administration of PART-derived tau to examine the effects of tau accumulation on microglial states separately from amyloid accumulation. Although other groups have reported that unique DAM populations seem to respond to tau accumulation (S. H. Lee et al., 2021; Kim et al., 2022), it is not easy to interpret whether the identified stage-specific microglia are really tau-related populations because the examined transgenic mice exhibited not only tau accumulation but also robust brain atrophy. Additionally, it remains unclear whether the frontotemporal-lobar-degeneration–causing mutations that are commonly used in tau transgenic mice actually recapitulate AD-related tau abnormalities. Furthermore, although aged model mice are usually used to produce substantial AD pathology, it is noteworthy that aging itself can lead to the emergence of a DAM-like population (Safaiyan et al., 2021). In the current study, we therefore used a combination of spatial transcriptomics to distinguish similar cell populations in single-cell RNA-seq data and found that tau accumulation increased DAM instead of WAM. Although it remains unclear whether and how WAM might exert their effects on tau pathology, it has been suggested that WAMs are involved in clearing myelin debris from the aged brain (Safaiyan et al., 2021). A decrease in WAM after tau accumulation may thus lead to adverse events, such as the accumulation of waste products, in the white matter of the brain.

There are some limitations to our study. First, we injected human PART-derived tau into dKI model mice only. Given that tau propagation and accumulation can be detected in WT mice after human PART tau injection (Ferrer et al., 2020), we speculate that accumulation and propagation of PART tau would be accelerated by the presence of amyloid plaques as well as by the humanization of tau protein, as shown in a previous study with AD tau (Saito et al., 2019). Further experiments with other mouse lines (i.e., WT, *App^NL-G-F^*, and *MAPT* knock-in mice) should therefore be performed to validate and strengthen our findings. Second, although we performed single-nucleus RNA-seq with a large number of cells three times independently, it remains unclear how and when the microglial states might change over time after tau accumulation. In addition, we cannot rule out the possibility that impurities during the extraction of tau from postmortem brains may have had unintended side effects on our experimental results. Furthermore, the exact spatial relationships between microglial subpopulations (such as DAM and WAM), amyloid-β, and tau remain unknown because of the limited resolution of our spatial transcriptomic analysis. Future work that discriminates the specific DAM populations in Tau-dKI brain sections at single-cell resolution will allow for a more appropriate interpretation of their involvement in tau pathology in the AD brain.

## Synthesis

Reviewing Editor: James Howard, Brandeis University Decisions are customarily a result of the Reviewing Editor and the peer reviewers coming together and discussing their recommendations until a consensus is reached. When revisions are invited, a fact-based synthesis statement explaining their decision and outlining what is needed to prepare a revision will be listed below. The following reviewer(s) agreed to reveal their identity: Li Fan. Note: If this manuscript was transferred from JNeurosci and a decision was made to accept the manuscript without peer review, a brief statement to this effect will instead be what is listed below.

The manuscript presents a compelling study on the role of tau pathology in altering microglial states in Alzheimer's disease (AD) model mice. The authors successfully developed a novel AD model by intracerebrally administering tau purified from human brains with primary age-related tauopathy into App knock-in mice with humanized tau. The study's key findings include the identification of Dectin-1-positive disease-associated microglia in response to tau accumulation and the demonstration that tau propagation and accumulation induce a disease-associated microglial phenotype at the expense of age-related white matter-associated microglia. These findings advance our understanding of how pathological forms of human tau may spread throughout the brain in the presence of A-beta deposition, and how p-tau spreading impacts the microglial response to A-beta. Although the mouse model is not fundamentally novel, it does incorporate some advances (using human PART Tau and a 3-/4-repeat tau knock-in) which may increase translational validity. The comments and suggestions listed below must be addressed before publication can be considered:

Major:

1. Image quantification is absent for Figures 1 and Extended Fig. 4. For Figure 1 A/B/D/E/F/G, the authors should quantify AT8 staining in ipsilateral vs contralateral hemisphere, ideally in several brain regions, for both timepoints, across at least 3 mice. NP Tau % area quantifications for panels C/D would also be useful. The replicability of the findings across several animals and with PART Tau from different sources is critical to assessing this mouse model.

2. Phosphorylated tau was highly distributed at both hippocampus and white matter at the inject side at 6 m.p.i while it is decreased in the hippocampus on the injection side after tau propagation at 12 m.p.i. The author should discuss the potential reason it is decreased at hippocampus but remains high at white matter.

3. One of the main goals of this paper is to "investigate the effects of tau accumulation on microglial cellular states in the brain", and yet, other than cell type frequency (Figure 3g), a direct comparison of Tau-dKI and dKI RNASeq data is missing. A package like DESeq2 or edgeR should be used to identify differentially expressed genes between Tau-dKI and dKI. Some form of pathway enrichment analysis would also be informative. A comparison with microglia RNAseq data from mouse models of tauopathy may also be considered.

4. There are issues of microgliosis quantification in Fig. 2, where it appears there are two different IBA1+ populations: IBA1+/Dectin-1+/Galectin-3+ triple positive microglia and IBA1+/Dectin-1-/Galectin-3- microglia. There are way more IBA1+ only microglia than triple positive microglia. This is not reflected in the quantification in Fig. 2C. Also, it appears that Dectin-1 and Calectin-3 are highly co-localized but not reflected in the last panel of Fig. 2B (Dectin-1&Galectin-3). Please address this apparent discrepancy.

5. Data in Extended Data Figure 4C should be quantified. For example, what fraction of microglia surrounding plaque express detectable levels of Ctnna3 or Dkk2 mRNA? How does this differ between dKI and Tau-dKI? Based on Figure 3a and 3b, Ctnna3 and Dkk2 are expressed in a very small percentage of DAMs based on the comparison with Cst7 (which is highly upregulated in most DAMs). Are Ctnna3 and Dkk2 labeling only a subset of DAMs, specifically DAM2 in this paper? Should Ctnna3 be referred to as "a novel DAM marker" (p.24) if it only labels <40% of DAMs?

6. The authors carefully examined Tau propagation and accumulation after human PART tau injection in dKI mice but not in WT mice, which potentially limits the interpretability of the findings. This potential limitation should be discussed by the authors in the manuscript, with reference to previous studies such as Saito et al. (JBC, 2019) in which comparisons between WT and dKI mice receiving tau injections were made.

Minor:

1. Panel labels in Figure 1 list panel D twice, therefore panel L is missing and the figure legend doesn't match the panels.

2. Figure 2C Y-axis labels are missing.

3. In Figure 2C, the authors should clarify whether Dectin-1-/Galectin-3- cells are identified as microglia based on Iba1 staining.

4. Upper case letters are used for panel labeling in Figure 1, but lower case letters are used for panel labeling in all other figures.

5. In the legend for Figure 3G, cluster 2 is labelled as "HAM". This abbreviation is never defined. It likely refers to homeostatic microglia, and should be abbreviated as "HM", as in extended figure 3. "HAM" refers to human Alzheimer's microglia in the literature and so it should not be used in this case.

6. Figure 3 abbreviations (eg. GM, WM, WAM, DAM etc.) should be defined in the figure legend.

7. Figure 3E heatmap is missing a scale bar. The scalebar should be labeled as "pseudotime".

8. Figure 3B heatmap coloring would be more easily interpretable if negative values are blue and positive values are red.

9. Fig. 3B. The authors should remove Tmeme119 from the dotplot as markers of microglia since it is lowly detected by snRNAseq compare to the other three markers used.

10. Fig. 3. Although oligodendrocytes are not the focus of the study, it is interesting for the readers to know the difference between cluster 0 and 3. The authors can add the top markers of cluster 0 and 3 to the dotplot. We recommend that markers of all clusters are added to the manuscript as a supplementary table.

## Author Response

Comments (Major points) Comment 1: Image quantification is absent for Figures 1 and Extended Fig. 4. For Figure 1 A/B/D/E/F/G, the authors should quantify AT8 staining in ipsilateral vs contralateral hemisphere, ideally in several brain regions, for both timepoints, across at least 3 mice. NP Tau % area quantifications for panels C/D would also be useful. The replicability of the findings across several animals and with PART Tau from different sources is critical to assessing this mouse model.

Response: Thank you very much for this important suggestion. We quantified the AT8 signals in ipsilateral and contralateral hippocampus, cerebral cortex, and corpus callosum of the PART tau-injected mice. AT8 signals were evaluated at both 6 m.p.i and 12 m.p.i. This quantification was performed in mice injected with PART tau from three different subjects (#8568, #8619, and #8739). We added this quantification data to Figure 1 (Fig.1 C-E). For NP tau, we tried to quantify positive areas in the model mouse brains according to a previous study (Nat Med 2018 PMID: 29200205). However, it was difficult to perform bias-free quantification because the accumulation of amyloid plaques was saturated and they were distributed throughout the sections of our 24-month-old model mice. Similarly, a previous study (Nature 2023 PMID: 37258678) has described the difficulty of reliably detecting amyloid plaques in AppNL-G-F knock-in mice where the amyloid plaque morphology was non-compacted compared with an overexpressing model (i.e. the 5XFAD line).

Comment 2: Phosphorylated tau was highly distributed at both hippocampus and white matter at the inject side at 6 m.p.i while it is decreased in the hippocampus on the injection side after tau propagation at 12 m.p.i. The author should discuss the potential reason it is decreased at hippocampus but remains high at white matter.

Response: Thank you for commenting on this critical point. Although we don't have a clear explanation for this observation, a substantial amount of phosphorylated tau is thought to be first generated in the injected side of the hippocampus. The phosphorylated tau then gradually propagates into wider brain regions. This generation around the injection site and transport to other regions might have resulted in the reduced signal in the hippocampus at 12 m.p.i. (Fig 1). A previous study has also reported that AT8 signals were reduced in the injected hippocampus at later stages (JEM 2016 PMID: 27810929). We added this description to the results section (p.16, line 7-10).

Comment 3: One of the main goals of this paper is to "investigate the effects of tau accumulation on microglial cellular states in the brain", and yet, other than cell type frequency (Figure 3g), a direct comparison of Tau-dKI and dKI RNASeq data is missing. A package like DESeq2 or edgeR should be 2 2 used to identify differentially expressed genes between Tau-dKI and dKI. Some form of pathway enrichment analysis would also be informative. A comparison with microglia RNAseq data from mouse models of tauopathy may also be considered.

Response: We appreciate the reviewer's suggestion. We newly searched for differentially expressed genes between Tau-dKI and dKI using the FindMarkers function in Seurat. Consistent with the cell type frequency result, we found a number of genes with altered expression in Tau-dKI (Extended Data Fig.

3-7). Several genes that showed dominant expression in microglial subcluster 0, such as Abca1 and Apobec1, were significantly downregulated in the Tau-dKI samples. In contrast, Ctnna3, a representative marker of microglial subcluster 3, was upregulated in the Tau-dKI samples. Using the identified gene lists, we performed enrichment analyses, and found that the enriched Gene Ontology terms were clearly different between the two samples (Extended Data Fig. 3-7). The additional experiments and the results have been added to the text (p.23, line 14-18; p.24 line 1-2). In addition, we reanalyzed the single-cell RNAseq dataset (Nat Commun 2022 PMID: 35413950) from P301S tauopathy mouse models to assess whether the identified marker genes could be detected in the tauopathy models. In contrast to the amyloid mouse models, the number of Cst7+ disease-associated microglia was limited in the tauopathy mice, which hampered comparison of the RNAseq data with our model (Extended Data Fig. 3-4). This result is described in the text (p.20, line 15-17).

Comment 4: There are issues of microgliosis quantification in Fig. 2, where it appears there are two different IBA1+ populations: IBA1+/Dectin-1+/Galectin-3+ triple positive microglia and IBA1+/Dectin-1-/Galectin-3- microglia. There are way more IBA1+ only microglia than triple positive microglia. This is not reflected in the quantification in Fig. 2C. Also, it appears that Dectin-1 and Calectin-3 are highly co-localized but not reflected in the last panel of Fig. 2B (Dectin-1&Galectin-3).

Please address this apparent discrepancy.

Response: First, we should apologize for the misleading image (old version of Fig. 2B) where the number of Dectin-1 &Galectin-3 double-positive microglia looked too low. In this image we added the IMARIS processed data, which presents the multicolor nature in a single color (for consideration for the visually impaired). This image processing resulted in the apparent discrepancy from the original confocal microscopy data. According to reviewer's critique, we replaced the processed IMARIS image with the original confocal data to match the quantification in Fig. 2C. We also recalculated the number of microglia in the confocal microscopy data using IMARIS to validate the quantification results. Although the values and the statistical significance were slightly altered, the tendency of an increased number of disease-associated microglia was confirmed. These added results are mentioned in the text (p.17-18).

3 3 Comment 5: Data in Extended Data Figure 4C should be quantified. For example, what fraction of microglia surrounding plaque express detectable levels of Ctnna3 or Dkk2 mRNA? How does this differ between dKI and Tau-dKI? Based on Figure 3a and 3b, Ctnna3 and Dkk2 are expressed in a very small percentage of DAMs based on the comparison with Cst7 (which is highly upregulated in most DAMs).

Are Ctnna3 and Dkk2 labeling only a subset of DAMs, specifically DAM2 in this paper? Should Ctnna3 be referred to as "a novel DAM marker" (p.24) if it only labels <40% of DAMs? Response: We appreciate the reviewer pointing this out. Although Ctnna3 signals could be detected in a substantial number of Iba1-positive microglia surrounding amyloid plaques, it was difficult to quantify the RNAscope data, not only because the signal to noise ratio was not high enough but also because Ctnna3 is probably dominantly expressed in oligodendrocytes. As the reviewer points out, the phrase "a novel DAM marker" might be inappropriate. We therefore corrected the relevant sentence to "Ctnna3 was partially expressed in DAM" to be more in line with the previous study and our reanalyzed Trem2 KO data (p21, line 7).

Comment 6: The authors carefully examined Tau propagation and accumulation after human PART tau injection in dKI mice but not in WT mice, which potentially limits the interpretability of the findings.

This potential limitation should be discussed by the authors in the manuscript, with reference to previous studies such as Saito et al. (JBC, 2019) in which comparisons between WT and dKI mice receiving tau injections were made.

Response: Thank you so much for this important suggestion. Although we only injected human PART tau into dKI mice, a previous study has shown that tau propagation and accumulation can be detected in WT mice after human PART tau injection (Brain Pathol. 2020 PMID: 31397930). Given that the conformation of PART tau is similar to that of AD tau, we speculate that accumulation and propagation of PART tau would be accelerated by the presence of amyloid plaque as well as by the humanization of tau protein as shown in a previous study (JBC 2019 PMID: 31273083). These sentences were added to the text (p26, line 10-15).

Comments (Minor points) Comment 1: Panel labels in Figure 1 list panel D twice, therefore panel L is missing and the figure legend doesn't match the panels.

Response: We have removed the duplicated letter D in the old version of Figure 1.

4 4 Comment 2: Figure 2C Y-axis labels are missing.

Response: We have labeled the Y-axis (Fig. 2C).

Comment 3: In Figure 2C, the authors should clarify whether Dectin-1-/Galectin-3- cells are identified as microglia based on Iba1 staining.

Response: We have labeled the Y-axis to clarify that Dectin-1−/Galectin-3− cells are identified as Iba1-positive microglia (Fig. 2C).

Comment 4: Upper case letters are used for panel labeling in Figure 1, but lower case letters are used for panel labeling in all other figures.

Response: We have unified the letter format to uppercase in all figures.

Comment 5: In the legend for Figure 3G, cluster 2 is labelled as "HAM". This abbreviation is never defined. It likely refers to homeostatic microglia, and should be abbreviated as "HM", as in extended figure 3. "HAM" refers to human Alzheimer's microglia in the literature and so it should not be used in this case.

Response: The mislabeled "HAM" was corrected to "HM".

Comment 6: Figure 3 abbreviations (eg. GM, WM, WAM, DAM etc.) should be defined in the figure legend.

Response: We added the relevant abbreviations to the figure legend (p. 37).

Comment 7: Figure 3E heatmap is missing a scale bar. The scalebar should be labeled as "pseudotime".

Response: We added a scale bar to the heatmap (Fig. 3E).

Comment 8: Figure 3B heatmap coloring would be more easily interpretable if negative values are blue and positive values are red.

Response: The heatmap coloring were appropriately changed.

Comment 9: Fig. 3B. The authors should remove Tmeme119 from the dotplot as markers of microglia since it is lowly detected by snRNAseq compare to the other three markers used.

Response: According to the reviewer's comment, we removed Tmem119 from the dotplot (Fig. 3B).

5 5 Comment 10: Fig. 3. Although oligodendrocytes are not the focus of the study, it is interesting for the readers to know the difference between cluster 0 and 3. The authors can add the top markers of cluster 0 and 3 to the dotplot. We recommend that markers of all clusters are added to the manuscript as a supplementary table.

Response: We newly explored the differentially expressed genes, but we did not discern any unique oligodendrocyte markers between the two oligodendrocyte clusters. The top five markers of cluster 0 contained Nav3, Cacna2d1, Cntn4, Dpyd, and Dscam, all of which showed higher expression in non-oligodendrocyte clusters (see Attached Figure 1 below). Therefore, we conclude that the difference between the two clusters was subtle and possibly caused by technical noise.

Also, according to the reviewer's suggestion, we added the marker information of all clusters to the manuscript as Extended Data Table 3-1.
