# Supplementary figures and images for: Tau Accumulation Induces Microglial State Alterations in Alzheimer's Disease Model Mice
Source: eNeuro. 2024 Dec 4;11(12):ENEURO.0260-24.2024. doi: 10.1523/ENEURO.0260-24.2024 (PMC11628182; doi:10.1523/ENEURO.0260-24.2024)

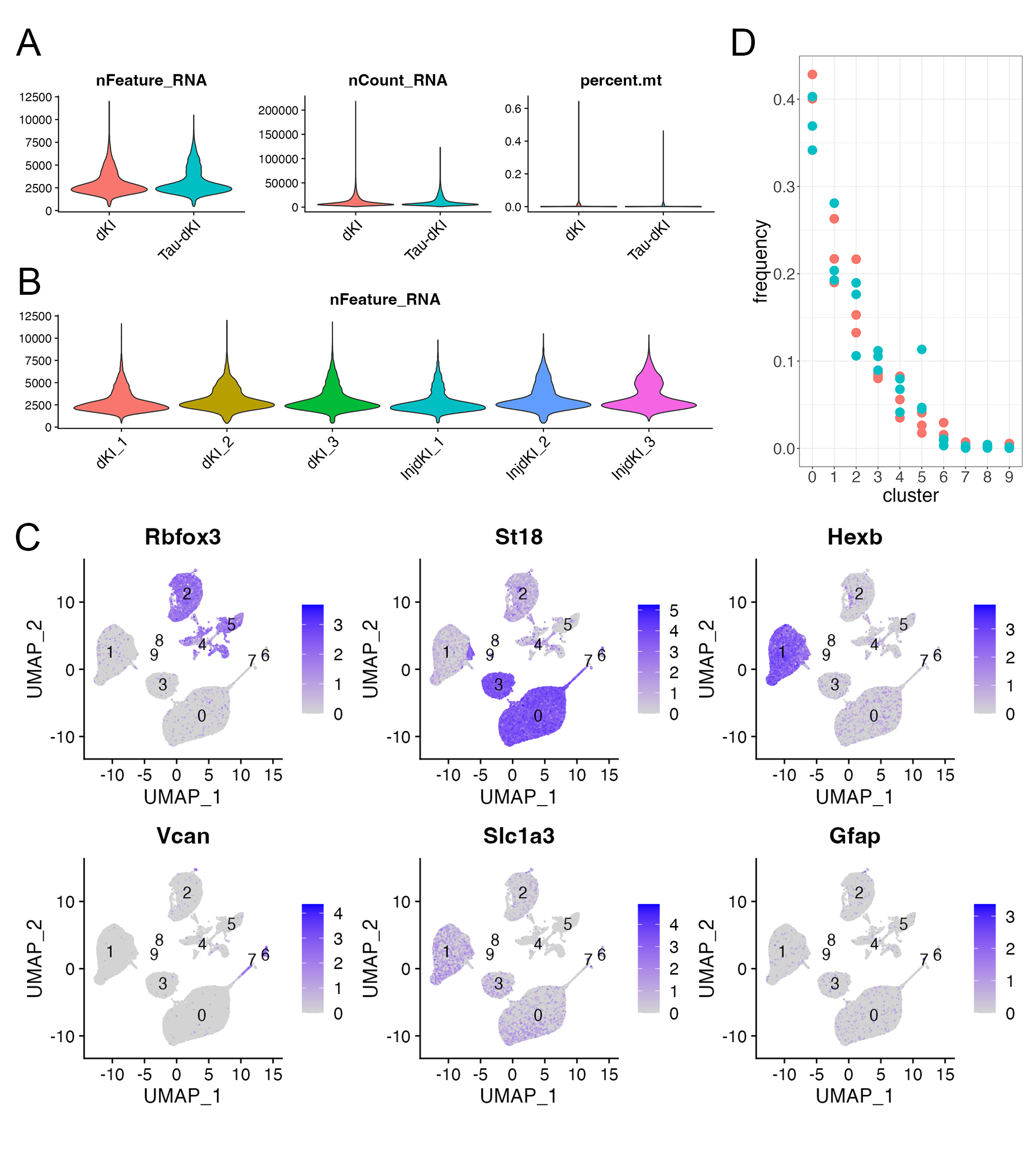

Supplement: Fig 3-1 — Basic information about the whole single-nucleus RNA-seq dataset A Violin plot showing the distributions of the number of detected genes, number of detected transcripts, and percentage of mitochondrial genes in each group. B Violin plot showing the distribution of the number of detected genes in each sample. C UMAP plots colored by the normalized expression levels of selected marker genes: Rbfox3 for neurons, St18 for oligodendrocytes, Hexb for microglia, Vcan for oligodendrocyte precursor cells, Slc1a3 for astrocytes, and Gfap for activated astrocytes. D Dot plot showing the frequencies of nuclei per cluster in dKI (red) and Tau-dKI (blue) mice. Download Fig 3-1, TIF file. [file eneuro-11-ENEURO.0260-24.2024-s001.tif]

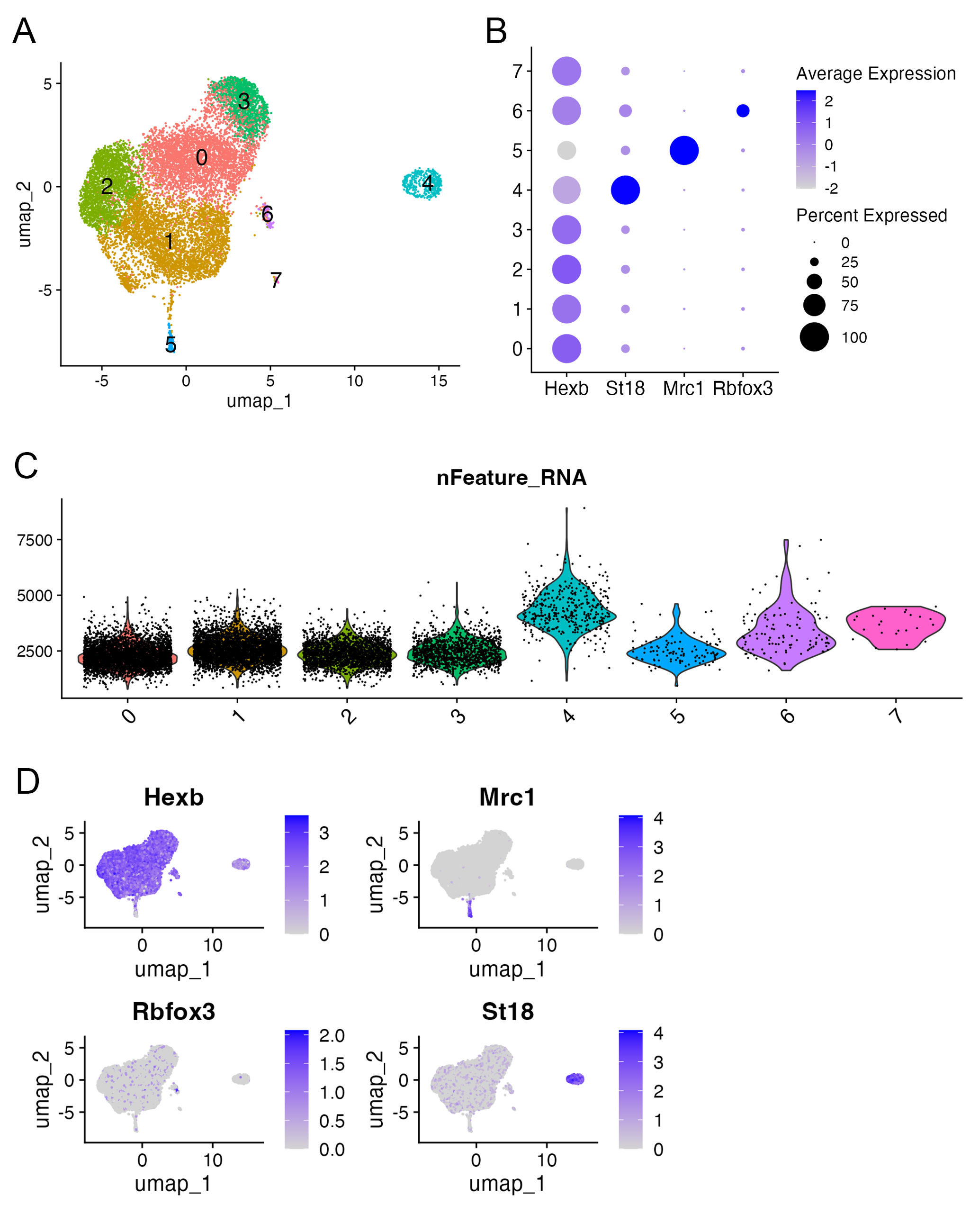

Supplement: Fig 3-2 — Characteristics of the Hexb+ population subtracted from the whole dataset A UMAP plot of the Hexb+ cluster (cluster 1 in Fig. 3) based on the recalculated scores. B Dot plot showing the average gene expression levels and the percentages of cells expressing marker genes across all clusters. Clusters 4 and 6 were likely doublet clusters because of their high expression of the non-microglial markers St18 and Rbfox3, respectively. Cluster 5 was evaluated to be a central nervous system border-associated macrophage because of the dominant expression of Mrc1. C Violin plots showing the distributions of the detected genes across the eight clusters. The average numbers of detected genes were relatively high in both clusters 4 (4,323 genes) and 6 (3,406 genes) compared with clusters 0–3 (2,422 genes). We also determined cluster 7 to be a homophilic doublet cluster because of the number of detected genes (3,628 genes). D UMAP plots, colored by the normalized expression levels of selected marker genes: Hexb for microglia, Mrc1 for central nervous system border-associated macrophages, Rbfox3 for neurons, and St18 for oligodendrocytes. Download Fig 3-2, TIF file. [file eneuro-11-ENEURO.0260-24.2024-s002.tif]

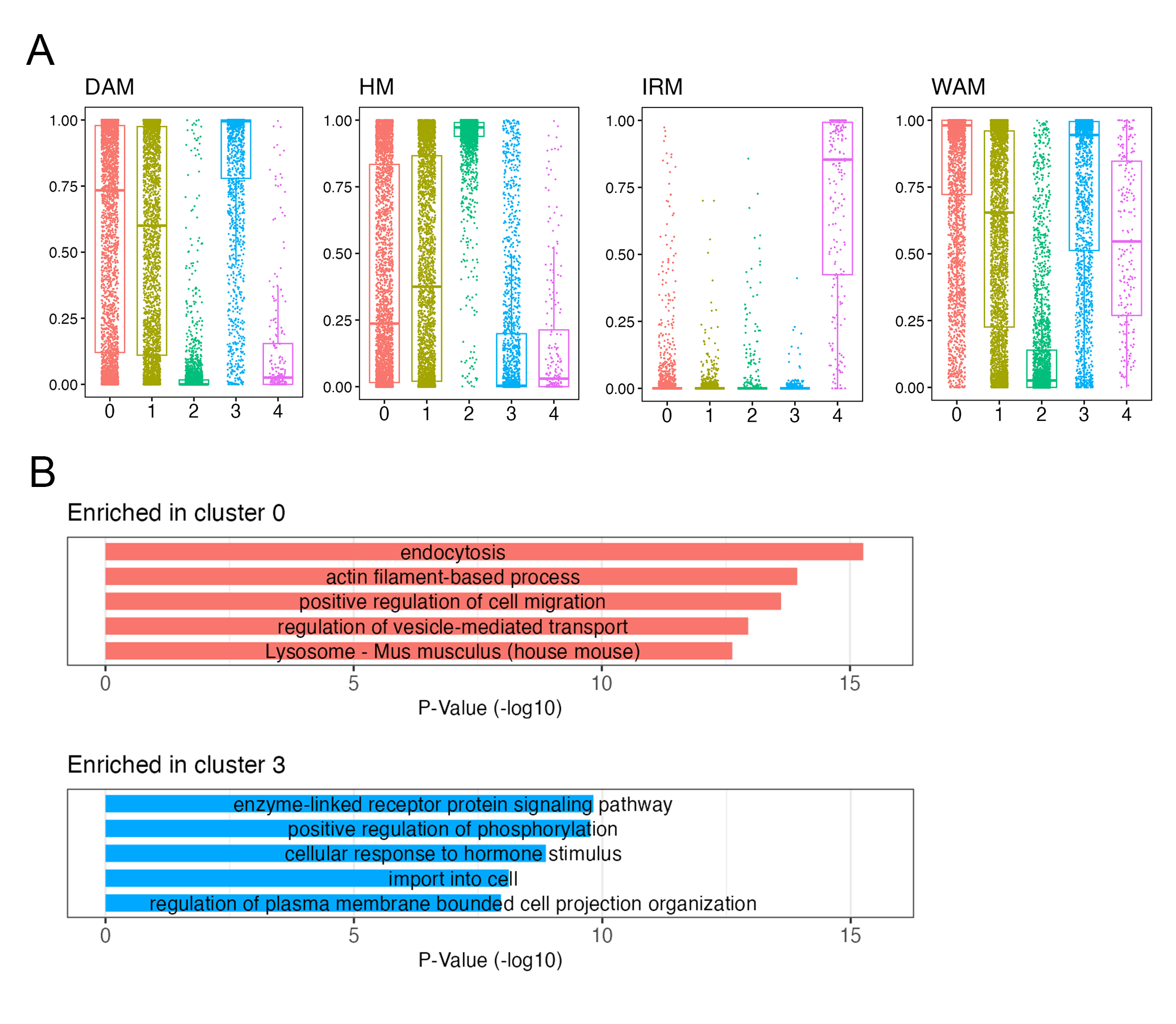

Supplement: Fig 3-3 — Characteristics of microglial subclusters A Similarity scores for the known microglial states in each cluster. We used sequence data to calculate similarity scores for DAM, homeostatic microglia (HM), interferon response microglia (IRM; all from accession number NCBI: GSE127893), and WAM (from accession number NCBI: GSE166548). B Gene Ontology terms associated with the genes enriched in microglial clusters 0 (upper) and 3 (lower). Enrichment analyses were performed using Metascape. Download Fig 3-3, TIF file. [file eneuro-11-ENEURO.0260-24.2024-s003.tif]

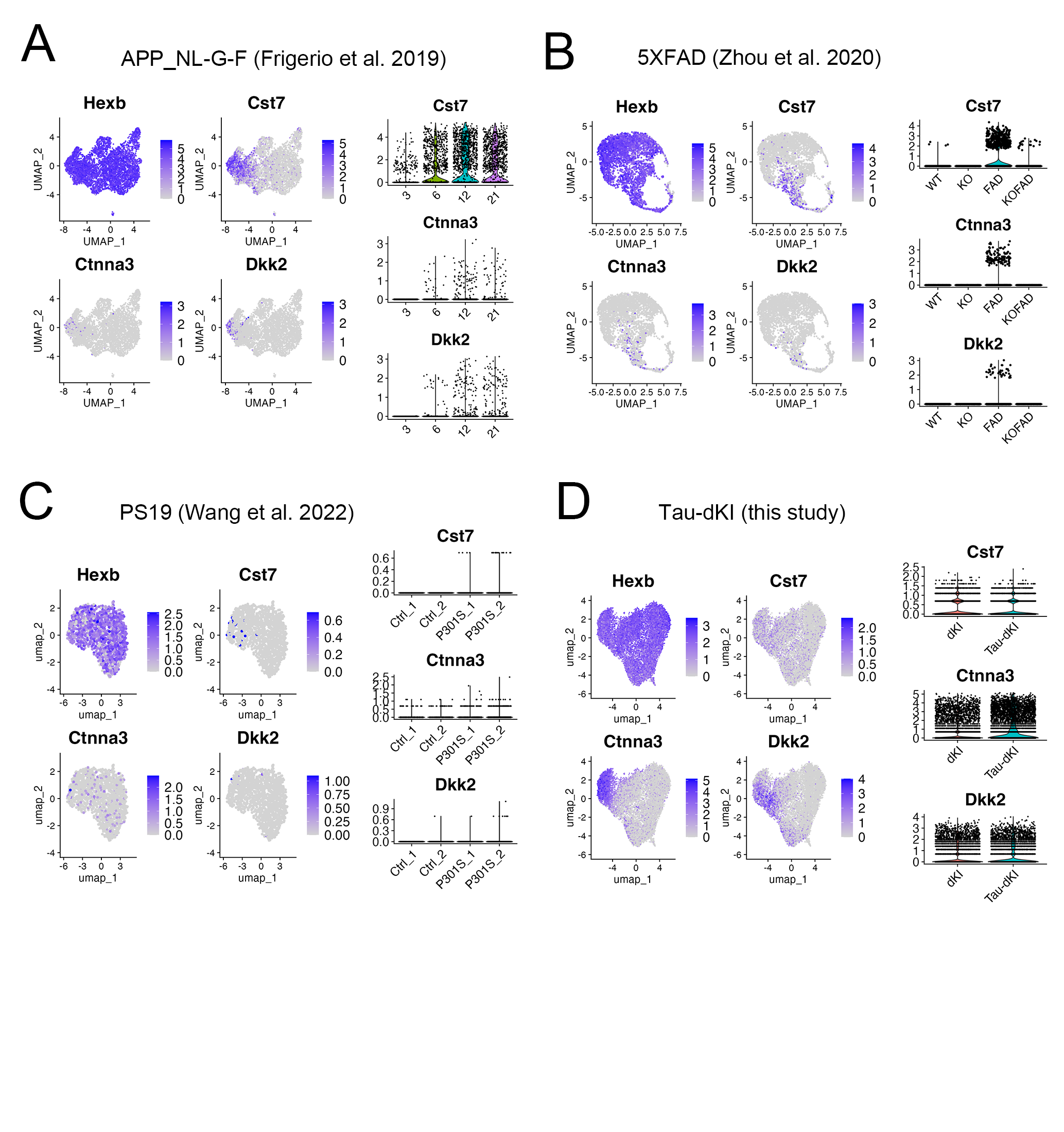

Supplement: Fig 3-4 — Ctnna3 expression in our Tau-dKI model and other AD models A (Left) UMAP plots colored by the normalized expression levels of selected genes. Hexb is a marker for microglia and Cst7 is a marker for DAM. (Right) Violin plots showing Cst7, Ctnna3, and Dkk2 expression in 3-, 6-, 12-, and 21-month-old AppNL-G-F mice. Sequence data from accession number NCBI: GSE127893. B (Left) UMAP plots colored by the normalized expression levels of selected genes. (Right) Violin plots showing Cst7, Ctnna3, and Dkk2 expression. Similar to known DAM markers, Ctnna3 expression was detected in 5XFAD microglia but not TREM2-deficient 5XFAD microglia. Sequence data from accession number NCBI: GSE140510. C (Left) UMAP plots colored according to the normalized expression levels of selected genes. (Right) Violin plots showing Cst7, Ctnna3, and Dkk2 expression. In contrast to known DAM markers, Ctnna3 expression was barely detected in P301S microglia. Sequence data from NCBI accession number: GSE198014. D (Left) UMAP plots colored according to the normalized expression levels of selected genes in Tau-dKI microglia. (Right) Violin plots showing Cst7, Ctnna3, and Dkk2 expression. Download Fig 3-4, TIF file. [file eneuro-11-ENEURO.0260-24.2024-s004.tif]

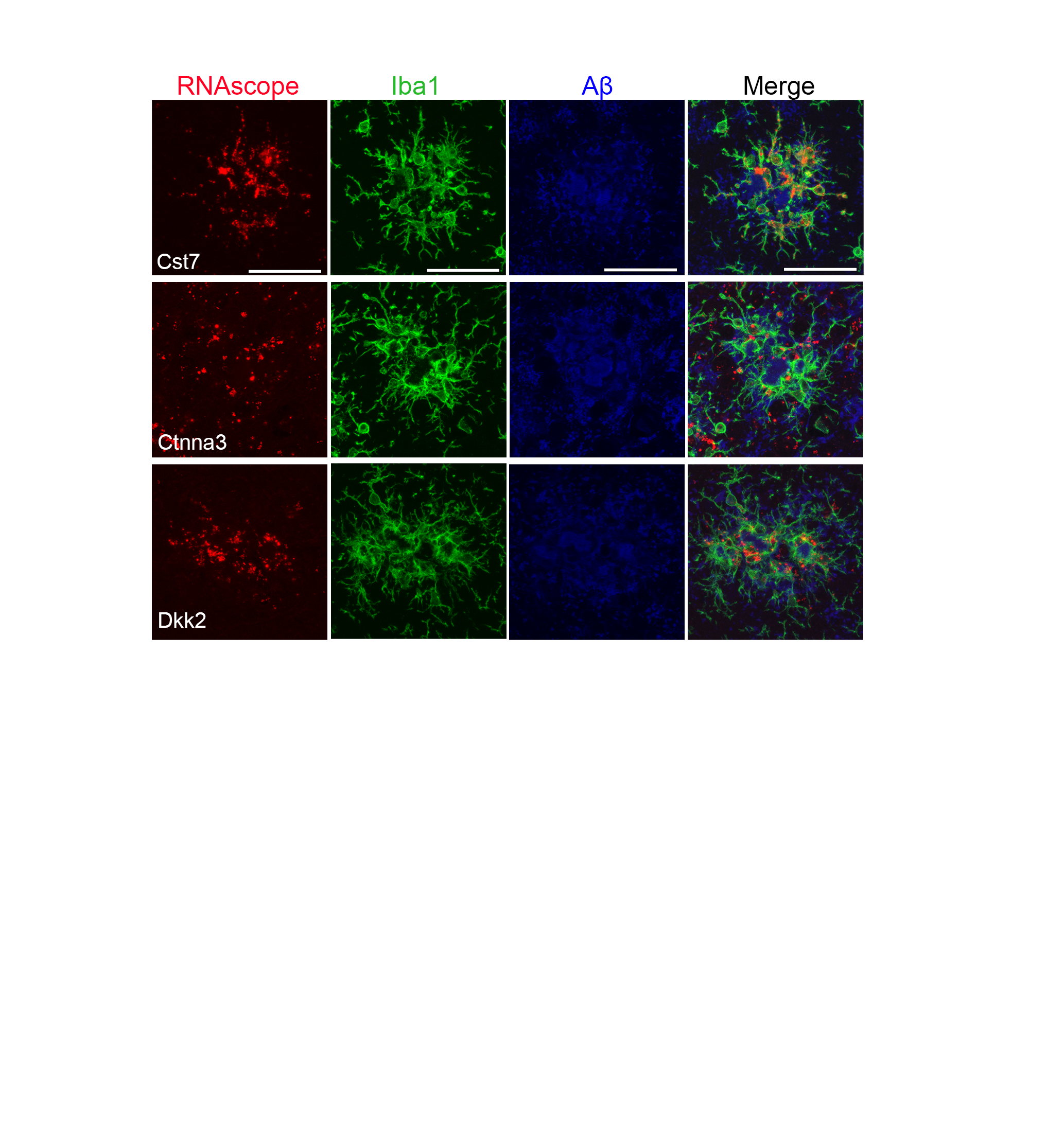

Supplement: Fig 3-5 — Histological distribution of Ctnna3 mRNA in aged AppNL-G-F brain Representative RNAscope images of in situ hybridization and immunohistochemical staining on 24-month-old AppNL-G-F brain sections, which were used to evaluate the spatial distribution of selected marker genes. Ctnna3 mRNA was partially colocalized with microglia surrounding amyloid-β. Scale bar: 50 μm. Download Fig 3-5, TIF file. [file eneuro-11-ENEURO.0260-24.2024-s005.tif]

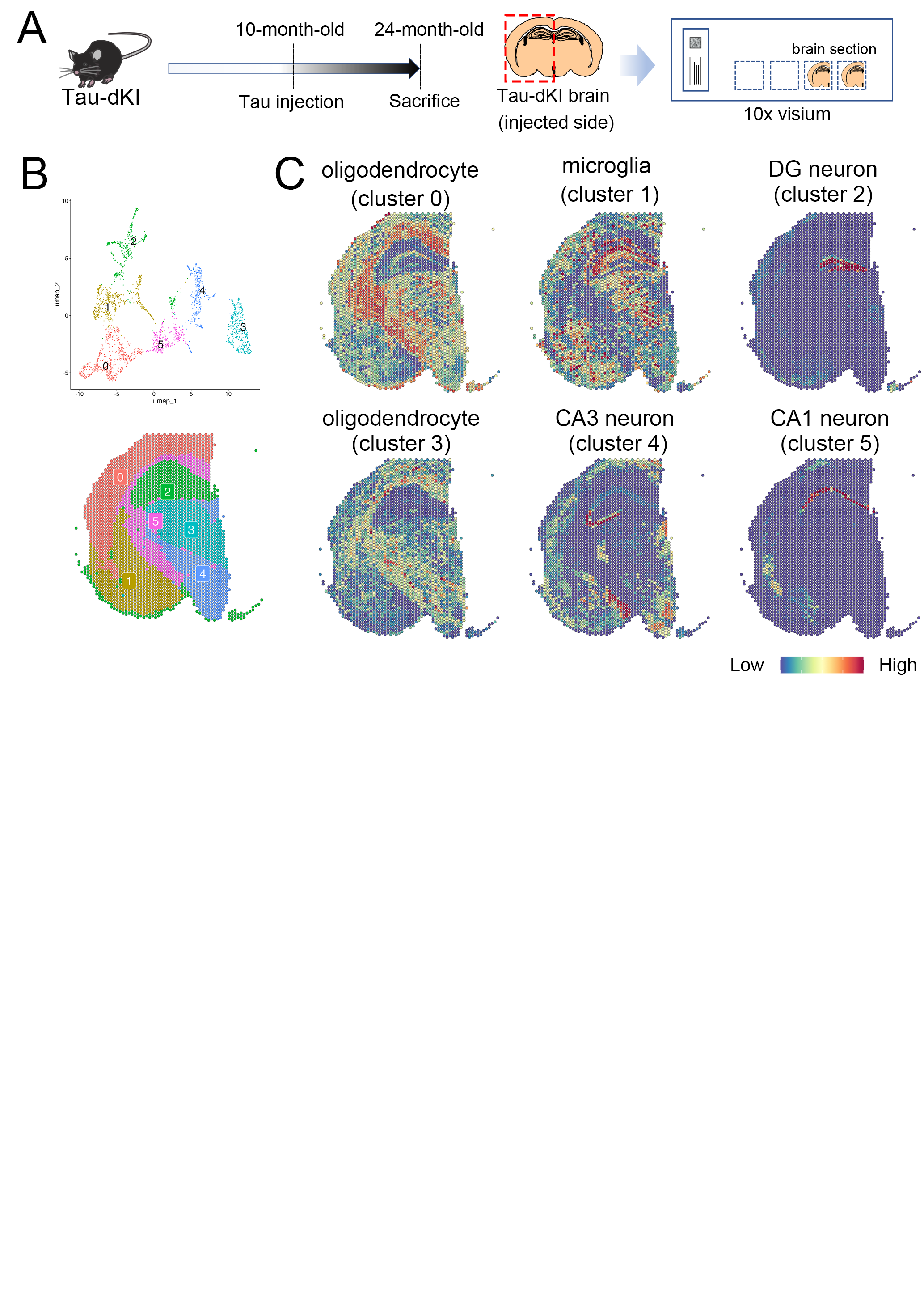

Supplement: Fig 3-6 — Spatial transcriptomics and integrative analyses with single-nucleus RNA-seq A Schematic diagram showing the experimental procedures of spatial transcriptomics. B UMAP plot of the 11,551 spots. C The six discriminated clusters corresponded to anatomical brain regions. D Integrative analyses of the spatial transcriptomics in a Tau-dKI brain section (injected side). The likelihood of the presence of each of the six clusters (0 to 5) at each spot on the section was estimated from single-nucleus RNA-seq data. St18 and Mog high oligodendrocyte clusters (0 and 3), Hexb+ microglia cluster (1), and Rbfox3+ neuronal clusters (2, 4, and 5). Download Fig 3-6, TIF file. [file eneuro-11-ENEURO.0260-24.2024-s006.tif]

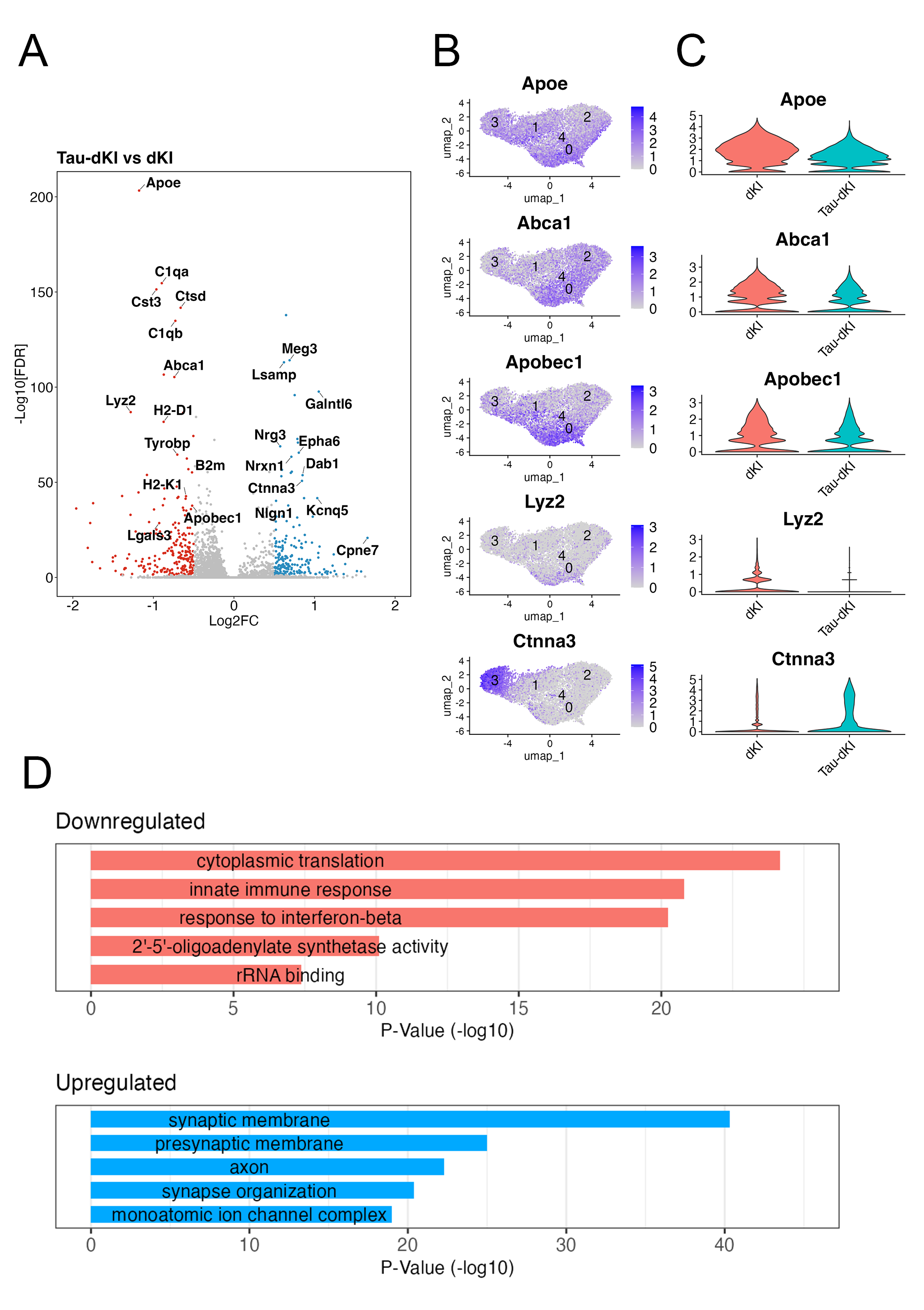

Supplement: Fig 3-7 — Differential expression analyses between Tau-dKI and dKI A Volcano plot showing differentially expressed genes identified by comparison of Tau-dKI vs. dKI. B UMAP plots colored according to the normalized expression levels of selected genes. C Violin plots showing the distributions of the detected genes in each group. D Gene Ontology terms associated with the enriched downregulated (upper) and upregulated (lower) genes in Tau-dKI. Enrichment analyses were performed using Metascape. Download Fig 3-7, TIF file. [file eneuro-11-ENEURO.0260-24.2024-s007.tif]
